# Development of pyrazoline-based derivatives as aminopeptidase N inhibitors to overcome cancer invasion and metastasis†

**DOI:** 10.1039/d1ra03629g

**Published:** 2021-06-17

**Authors:** Jiangying Cao, Chunlong Zhao, Hang Dong, Qifu Xu, Yingjie Zhang

**Affiliations:** Department of Medicinal Chemistry, Key Laboratory of Chemical Biology (Ministry of Education), School of Pharmaceutical Sciences, Cheeloo College of Medicine, Shandong University 44 West Wenhua Road Jinan Shandong 250012 P. R. China zhangyingjie@sdu.edu.cn +86 531 88382009 +86 531 88382009; School of Pharmacology, Shandong University of Traditional Chinese Medicine Jinan 250355 P. R. China

## Abstract

Aminopeptidase N is considered as a promising anti-tumor target due to its role in tumor invasion, metastasis and angiogenesis. In this report, a new series of pyrazoline-based derivatives were designed, synthesized and evaluated for biological activities. The structure–activity relationships of these pyrazoline-based derivatives were also discussed in detail. Among them, compound 2k, with 2,6-dichloro substitution, showed the best APN inhibitory activity, of which the IC_50_ value was two orders of magnitude lower than that of the positive control bestatin. At the same concentration of 100 μM, the *in vitro* anti-invasion activity of compound 2k was also significantly better than that of bestatin. Moreover, compound 2k could effectively prevent the pulmonary metastasis of mice H22 hepatoma cells *in vivo*, supporting its further research and development as an antitumor agent.

## Abstract 2

Compound 2k exhibited promising *in vitro* anti-invasion and *in vivo* anti-metastasis potencies, suggesting its prospect as an anti-invasion and anti-metastasis lead.

## Graphical abstract

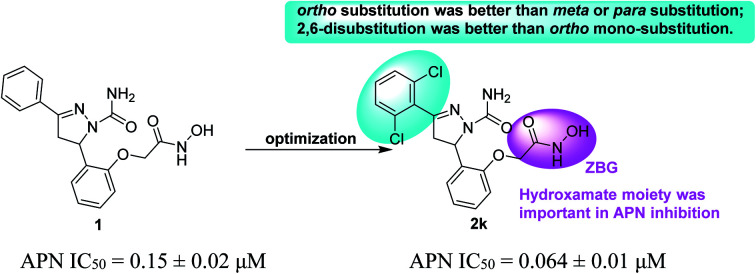

## Introduction

Aminopeptidase N (APN, CD13), a type II glycoprotein, is a member of the superfamily of the zinc-dependent M1 aminopeptidase.^1,2^ APN is mainly found in liver, the renal brush border, intestines, epithelial cells, endothelial cells and so on.^3^ As an exopeptidase, the enzyme preferentially hydrolyzes the neutral or basic amino acid residues from the N-terminals of oligopeptides.^4^ Besides cleaving biological peptides, APN also functions as a signaling molecule and a receptor of coronavirus, so consequently is designated to be a “moonlighting enzyme”.^5^ Enhanced APN expression was observed in many tumor cells, such as melanoma, colon, thyroid, renal, breast, lung and liver cancers, and was associated with poor prognosis.^6^ Metastasis is a main cause of chemotherapeutical failure and tumor recurrence. Basement membrane is the main barrier of the invasion and metastasis of tumor. APN could hydrolyze the type-IV collagen, the main ingredient of basement membrane, and promote tumor metastasis.^7^ Moreover, compared with the normal vessels, up-regulated APN expression is exclusively found in the angiogenic vasculatures.^8^ The cooperative effects of APN on both tumor cells and nonmalignant stromal cells within the tumor microenvironment promoted the angiogenesis, in response to hypoxia or angiogenic growth factor stimulation.^9^ Impaired neovascularizations were found in APN-null mice.^10^ What's more, APN was considered as one member of surface markers of liver cancer stem cells.^11^ The combination of APN inhibitor bestatin and cytotoxic drug 5-Fu demonstrated synergistic anti-hepatoma effect, by inhibiting hepatoma stem cells and normal hepatoma cells, respectively.^12^ All these findings support the rationale that APN is a promising antitumor target.

**Fig. 1:**
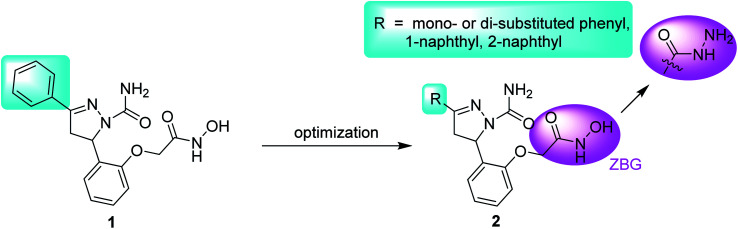
The design strategy of novel APNIs.

Structurally, APN, composed of 967 amino acids, can be divided into four parts: a short intracellular tail, a transmembrane anchor, a small serine-/threonine-rich extracellular stalk and a large ectodomain. The X-ray crystal structure of APN-bestatin complex revealed that the catalytic site existing in the ectodomain contained a catalytic zinc ion and three hydrophobic pockets (S_1_, S′_1_ and S′_2_).^13,14^ To date, many natural products and synthetic small molecules were reported as APN inhibitors (APNIs). Natural products include bestatin,^15^ probestin,^16^ amastatin,^17^ AHPA-Val,^18^ and so on. Synthetic APNIs contain amino-benzosuberone derivative,^19^ flavone derivative,^20^ aminophosphinic derivative,^21^ ureido peptidomimetics,^22^ and so on. The structure–activity relationships (SARs) of potent APNIs revealed that zinc binding group (ZBG) and hydrophobic group interacting with at least one of three hydrophobic pockets were essential for the activities against APN.^23^ In our previous work, compound 1, one pyrazoline-based hydroxamate derivative, exhibited much more potent inhibitory activity against APN than the positive control bestatin.^24^ In order to extend the SARs of this series of compounds and find more potent compounds, derivatization was focused on the terminal phenyl group of compound 1, leading to compound 2 (Fig. 1). In the structure of compound 2, the introduction of the hydrophobic substituents on the terminal phenyl group or the replacement of the phenyl group with naphthyl group may enhance the hydrophobic interaction with APN.^25^ Besides, one compound with the hydrazide group was also synthesized to investigate the effect of the ZBG on APN inhibition.

## Results and discussion

### Chemistry

**Scheme 1:**
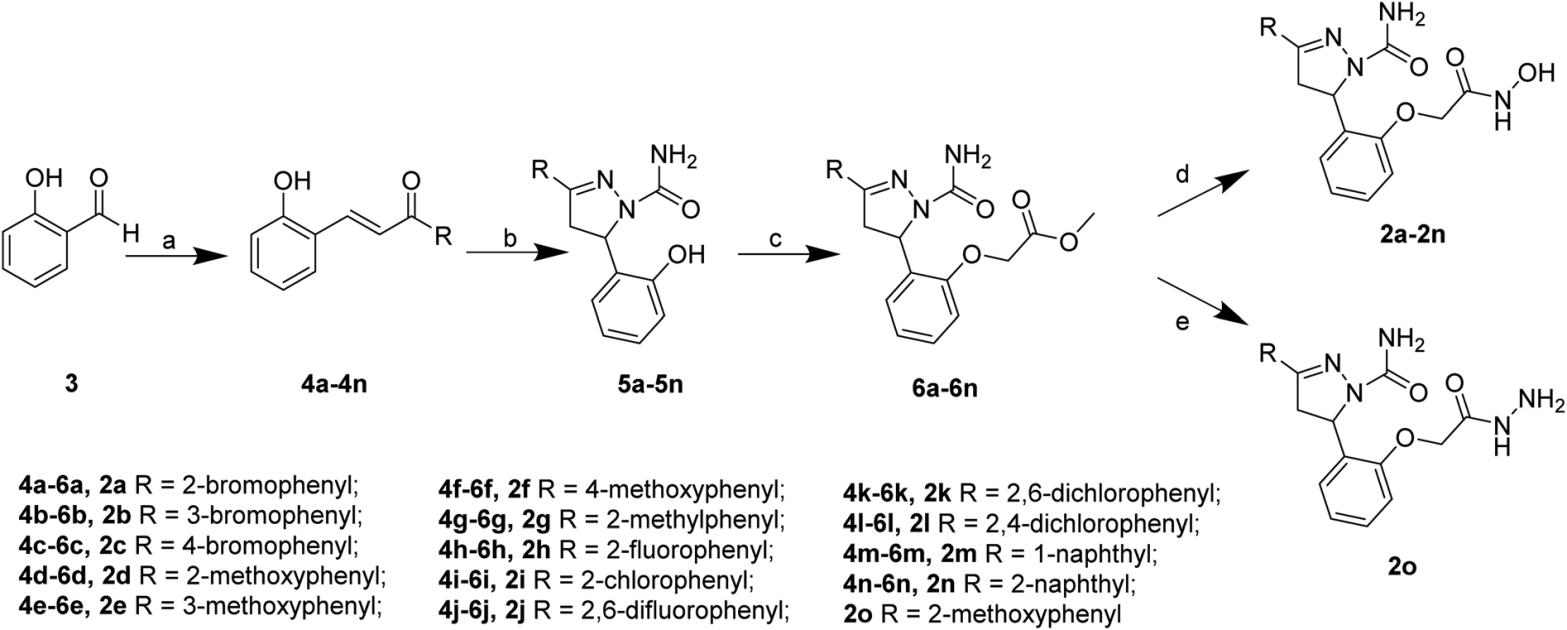
Reagents and conditions: (a) substituted acetophenone for 4a–4l, 1-(naphthalen-1-yl)ethan-1-one for 4m, 1-(naphthalen-2-yl)ethan-1-one for 4n, KOH, 80% EtOH, 25 °C, 48 h; (b) semicarbazide hydrochloride, NaOH, EtOH, 78 °C, 5 h; (c) DMF, NaH, methyl bromoacetate, 25 °C, 12 h; (d) NH_2_OK, MeOH, 25 °C, 0.5 h; (e) 6d, 80% hydrazine hydrate, MeOH, 65 °C, 6 h.

The target compounds 2 were synthesized according to the procedures listed in Scheme 1. Firstly, 2-hydroxybenzaldehyde 3 reacted with corresponding ketone to give the chalcone derivatives 4 by the Claisen–Schmidt condensation. Subsequently, compounds 4 underwent cyclization with semicarbazide hydrochloride to form the pyrazoline-based intermediates 5, which further reacted with methyl bromoacetate to give compounds 6. Finally, the methyl ester groups of 6 were transformed to hydroxamate groups by NH_2_OK to give target compounds 2a–2n. Besides, compound 6d reacted with hydrazine hydrate led to compound 2o.

### Inhibitory activities of the target compounds against APN

All the newly synthesized pyrazoline-based derivatives were firstly evaluated for their inhibitory activities against APN using bestatin and compound 1 as the positive controls. The results are listed in Table 1. Compound 2o with the hydrazide group was over-100 fold less potent than its corresponding hydroxamate analog 2d, indicating the important role of the hydroxamate group as ZBG in APN inhibition. Comparing compounds 2a–2f, it was easily observed that compounds with mono-substituent in the *ortho* position exhibited better activities against APN than the counterparts with mono-substituent in the *meta* or *para* position (2a*vs.*2b, 2c; 2d*vs.*2e, 2f). Therefore, compounds (2g–2i) with various mono-substituents in the *ortho* position were synthesized and evaluated. Among the *ortho* mono-substituted analogs (2a, 2d, 2g–2i), compound 2i with *ortho* chlorine substitution exhibited the best APN inhibitory activity, with the IC_50_ value of 0.10 ± 0.01 μM. The inhibitory order of compounds containing *ortho* mono-substituents was 2i (2-Cl) > 2g (2-CH_3_) > 2f (2-F), 2a (2-Br) > 2d (2-OCH_3_), suggesting that the hydrophobicity and size of substituents both impact the inhibitory activities against APN. Moreover, compounds 2j–2l were synthesized to explore the effects of dual-substituents of the phenyl group on APN inhibition. Compound 2k with 2,6-dichlorine substitution showed better APN inhibitory activity than the counterparts 2j and 2l, which possessed 2,6-difluorine substitution and 2,4-dichlorine substitution, respectively. Moreover, 2,6-dual substituted analogues 2j and 2k showed slightly better APN inhibition than the *ortho* mono-substituted counterparts 2h and 2i, respectively. Replacement of the terminal phenyl group of compound 1 using naphthyl group led to compounds 2m and 2n. Compound 2m with 1-naphthyl group was over 10-fold more potent than 2n with 2-naphthyl group. However, the APN inhibitory activity of 2m was inferior to those of compounds with terminal phenyl group, including compounds 1, 2a and 2g–2k. Satisfactorily, compounds 2a and 2g–2k exhibited comparable or improved APN inhibitory activities relative to the parent compound 1, supporting our design strategy. Notably, the most potent one 2k (IC_50_ = 0.064 ± 0.01 μM) was over 2-fold more potent than compound 1 (IC_50_ = 0.15 ± 0.02 μM) and over 140-fold more potent than the positive control bestatin (IC_50_ = 9.0 ± 0.5 μM).

**Table 1:** The structures and IC_50_ values of target compounds against porcine APN

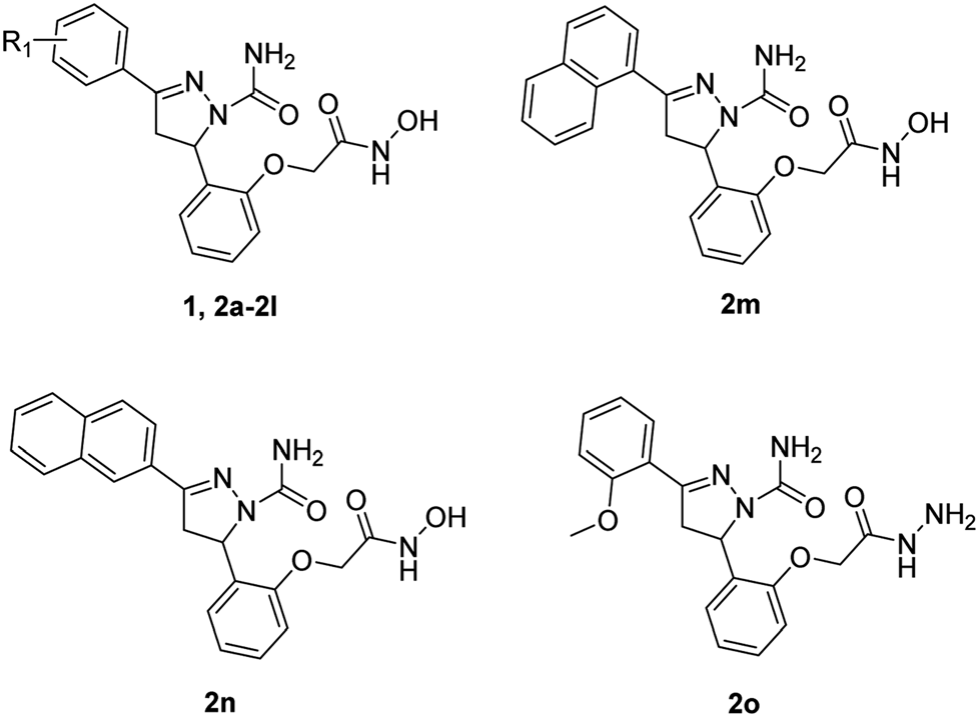		
Compd	R_1_	APN IC_50_ (μM)^a^
1	H	0.15 ± 0.02
2a	2-Br	0.15 ± 0.03
2b	3-Br	0.26 ± 0.05
2c	4-Br	2.1 ± 0.2
2d	2-OCH_3_	0.27 ± 0.01
2e	3-OCH_3_	0.32 ± 0.03
2f	4-OCH_3_	12.5 ± 0.8
2g	2-CH_3_	0.11 ± 0.01
2h	2-F	0.15 ± 0.01
2i	2-Cl	0.10 ± 0.01
2j	2,6-diF	0.12 ± 0.01
2k	2,6-diCl	0.064 ± 0.01
2l	2,4-diCl	0.42 ± 0.03
2m	—	0.21 ± 0.04
2n	—	2.5 ± 0.1
2o	—	30.2 ± 1.9
Bestatin	—	9.0 ± 0.5

a Assays were performed in triplicate; data are shown as mean ± SD.

### Inhibitory activities of 2k against the proliferation of tumor cells *in vitro*

Compound 2k with the best APN inhibitory activity was selected for further anti-proliferative evaluation against six tumor cell lines (U937, K562, PLC/PRF/5, PC-3, ES-2, HepG2). The results are listed in Table 2. Though better than bestatin, compound 2k showed only moderate anti-proliferative activities against the selected tumor cell line, indicating the low cytotoxicity of 2k against tumor cells.

**Table 2:** *In vitro* anti-proliferative activities of selected compounds

Compd	IC_50_ (μM)^a^					
	U937	K562	PLC/PRF/5	PC-3	ES-2	HepG2
2k	49.2 ± 3.8	15.6 ± 2.2	105.5 ± 4.7	56.1 ± 3.1	141.3 ± 3.8	101.4 ± 4.9
Bestatin	>500	>500	>500	>500	>500	>500

a Assays were performed in triplicate; data are shown as mean ± SD.

### Anti-invasion assay *in vitro*

**Fig. 2:**
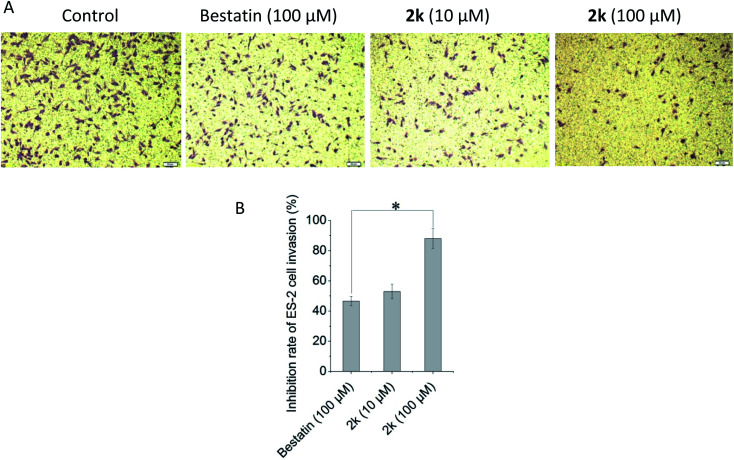
Effect of compound 2k against ES-2 cell invasion. (A) Representative images of ES-2 cell invasion treated with bestatin or compound 2k. (B) The inhibition rates of tested compounds against ES-2 cells invasion. Data are expressed as the mean ± standard deviation for triplicate experiments. *P < 0.05, 2k (100 μM) *vs.* bestatin (100 μM).

APN could hydrolyze the basement membrane to promote the invasion and metastasis of tumor cells. In this research, the transwell chambers coated with matrigel that could mimic the basement membrane were used to evaluate the anti-invasion effects of selected compounds *in vitro*. As shown in Fig. 2, compound 2k could inhibit the ES-2 cells invasion in a concentration dependent manner. It was remarkable that 10 μM of 2k exhibited comparable, if not better, anti-invasion activity than that of 100 μM of bestatin. At the same concentration of 100 μM, the anti-invasion effect of 2k was significantly better than that of bestatin. Note that the compound concentrations used in anti-invasion assay were lower than the IC_50_ values against ES-2 cells shown in Table 2, confirming the anti-invasion effects were not due to cytotoxicity against ES-2 cells.

### Anti-metastasis assay *in vivo*

**Fig. 3:**
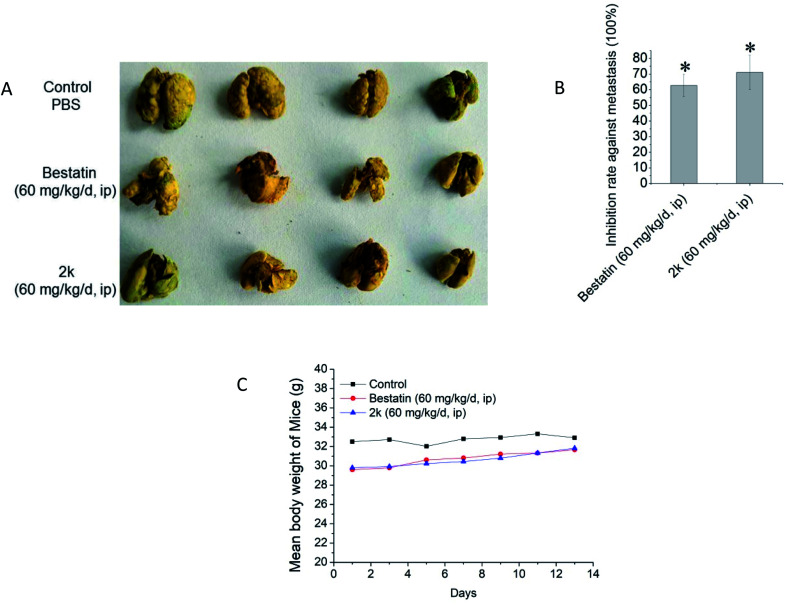
Anti-metastasis potency of tested compounds using mice H22 hepatoma cell pulmonary metastasis model. (A) Picture of pulmonary lobes with metastasis nodes. (B) Inhibition rate of the tested compounds against metastasis. Data are expressed as mean ± SD. *P < 0.05, treated group *vs.* control group. (C) Mean body weights of mice monitored every two days.

Encouraged by the *in vitro* anti-invasion effect of 2k, we further evaluated the *in vivo* anti-metastasis effect of 2k using the mice H22 hepatoma cell pulmonary metastasis model. The metastatic nodes on the surface of pulmonary lobes were counted and the inhibition rate was calculated. As shown in Fig. 3, at the dose of 60 mg kg^−1^ d^−1^, bestatin and 2k demonstrated similar *in vivo* anti-metastasis effects, both of which could obviously decrease the numbers of pulmonary lobes. The potent *in vivo* anti-metastasis effect of 2k was in line with its potent *in vitro* anti-invasion activity shown in Fig. 2. Besides, in the mice groups treated by bestatin and 2k, no significant body weight loss was observed (Fig. 3C), suggesting no systematic toxicities, which were in line with the marginal *in vitro* cytotoxicity against tumor cells shown in Table 2. All experiments involving laboratory animals were performed with the approval by the institutional guidelines of Animal Care and Use Committee at Shandong University.

## Conclusion

In summary, a new series of APN inhibitors with pyrazoline scaffold was designed, synthesized and evaluated. In the enzymatic assay against APN, except 2f, all the compounds with the hydroxamate moiety displayed better activities than the positive control bestatin. Satisfactorily, compounds 2a, 2g, 2h, 2i, 2j and 2k showed similar or better APN inhibitory activities relative to their lead compound 1. Moreover, compound 2k with the best APN inhibitory activity exhibited promising *in vitro* anti-invasion and *in vivo* anti-metastasis potencies, suggesting its prospect as anti-invasion and anti-metastasis lead.

## Materials and methods

### Chemistry: general procedures

All the commercially available materials were used without further purification otherwise noted. All reactions were monitored by thin-layer chromatography (TLC) on 0.25 mm silica gel plates (60 GF-254) and the product spots were visualized by UV light, ferric chloride and iodine vapor. The purification of the products was conducted by column chromatography and recrystallization. Melting points determined on an electrothermal melting point apparatus were not corrected. Using TMS as an internal standard, ^1^H-NMR and ^13^C-NMR spectra were determined on a Brucker DRX spectrometer and the values of chemical shifts were described as *δ* in parts per million and *J* in Hertz. HRMS were conducted by Shandong Analysis and Test Center.

### (*E*)-1-(2-Bromophenyl)-3-(2-hydroxyphenyl)prop-2-en-1-one (4a)

To a solution of compounds 3 (6.10 g, 50 mmol) and 1-(2-bromophenyl)ethan-1-one (11.88 g, 60 mmol) in ethanol (200 mL) was added potassium hydroxide aqueous solution (25.20 g, 450 mmol, in 50 mL water) at 0 °C. After stirring at 25 °C for 48 h, the mixture was poured into ice-cold water and 1N HCl solution was added to neutralize the excess base. The formed precipitate was filtered off and further purified by column chromatograph (DCM/MeOH = 100 : 5) to give 8.61 g of compound 4a as a yellow solid. Yield: 57%, mp: 140.2–142.2 °C. ^1^H NMR (400 MHz, DMSO-*d*_*6*_): *δ* 10.33 (s, 1H), 7.75 (d, *J* = 7.8 Hz, 1H), 7.70 (d, *J* = 7.8 Hz, 1H), 7.61 (d, *J* = 16.3 Hz, 1H), 7.56–7.50 (m, 2H), 7.49–7.44 (m, 1H), 7.29 (t, *J* = 8.5 Hz, 1H), 7.20 (d, *J* = 16.3 Hz, 1H), 6.92 (d, *J* = 7.8 Hz, 1H), 6.86 (t, *J* = 7.5 Hz, 1H).

Compounds 4b–4n were prepared in a similar manner as described for compound 4a.

### 3-(2-Bromophenyl)-5-(2-hydroxyphenyl)-4,5-dihydro-1*H*-pyrazole-1-carboxamide (5a)

To a solution of 4a (6.04 g, 20 mmol) in ethanol (100 mL) was added semicarbazide hydrochloride (2.68 g, 24 mmol) and sodium hydroxide (3.60 g, 90 mmol). After stirring at 78 °C for 5 h, water (100 mL) was added into the mixture and 10% HCl solution was used to adjust pH to 6. The white solid formed from the mixture was filtered off and washed with EtOAc to give 4.67 g of compound 5a. Yield: 65%, mp: 218.4–220.2 °C. ^1^H NMR (400 MHz, DMSO-*d*_6_): *δ* 10.01 (s, 1H), 7.70–7.68 (m, 2H), 7.43 (td, *J* = 7.6 Hz, *J* = 1.3 Hz, 1H), 7.33 (td, *J* = 7.7 Hz, *J* = 1.8 Hz, 1H), 7.02 (td, *J* = 7.7 Hz, *J* = 1.7 Hz, 1H), 6.89 (dd, *J* = 7.6 Hz, *J* = 1.7 Hz, 1H), 6.80 (dd, *J* = 8.1 Hz, *J* = 1.2 Hz, 1H), 6.67 (t, *J* = 7.4 Hz, 1H), 6.46 (s, 2H), 5.56 (dd, *J* = 11.9 Hz, *J* = 4.8 Hz, 1H), 3.91 (dd, *J* = 17.7 Hz, *J* = 11.9 Hz, 1H), 3.04 (dd, *J* = 17.7 Hz, *J* = 4.8 Hz, 1H).

Compounds 5b–5n were prepared in a similar manner as described for compound 5a.

### Methyl 2-(2-(3-(2-bromophenyl)-1-carbamoyl-4,5-dihydro-1*H*-pyrazol-5-yl) phenoxy)acetate (6a)

To a solution of compound 5a (3.59 g, 10 mmol) in DMF (30 mL) was added 60% sodium hydride (0.48 g, 12 mmol) gradually. The mixture was stirred at 25 °C for 5 minutes and methyl bromoacetate (2.26 g, 15 mmol) was added. After stirring at 25 °C for 12 h, the mixture was poured into ice-cold water. Then the mixture was extracted with EtOAc (100 mL × 3), washed with brine (50 mL × 3) and dried over MgSO_4_. Filtration and concentration gave the crude product, which was further purified by column chromatography (DCM/MeOH) to give 2.80 g of compound 6a as a white solid. Yield: 65%, mp: 144.4–146.2 °C. ^1^H NMR (400 MHz, DMSO-*d*_6_): *δ* 7.71–7.66 (m, 2H), 7.44 (t, *J* = 7.6 Hz, 1H), 7.34 (t, *J* = 7.6 Hz, 1H), 7.21 (t, *J* = 7.6 Hz, 1H), 7.01–6.92 (m, 3H), 6.50 (s, 2H), 5.63 (dd, *J* = 12.0 Hz, *J* = 5.0 Hz, 1H), 4.93–4.85 (m, 2H), 3.94 (dd, *J* = 17.8 Hz, *J* = 12.0 Hz, 1H), 3.68 (s, 3H), 3.10 (dd, *J* = 17.8 Hz, *J* = 5.0 Hz, 1H).

Compounds 6b–6n were prepared in a similar manner as described for compound 6a.

### 3-(2-Bromophenyl)-5-(2-(2-(hydroxyamino)-2-oxoethoxy)phenyl)-4,5-dihydro-1*H*-pyrazole-1-carboxamide (2a)

Potassium hydroxide (28.00 g, 509 mmol) was dissolved in distilled methanol (70 mL) at 0 °C. The mixture was added into hydroxylamine hydrochloride (23.35 g, 343 mmol) in distilled methanol (120 mL) gradually. Then the mixture was stirred at 0 °C for 40 min. The formed precipitate was filtered out to give a fresh methanol solution of potassium hydroxylamine as the effective agent for hydroxamate formation. Compound 6a (0.86 g, 2 mmol) was dissolved in the above solution (8 mL). After stirring at 25 °C for 0.5 h, the mixture was poured into water (50 mL) and 10% HCl solution was added to adjust pH to 6. The formed precipitate was filtered off and dried under vacuum to give 0.45 g of compound 2a. White solid, yield: 52%, mp: 168.4–170.2 °C. ^1^H NMR (400 MHz, DMSO-*d*_6_): *δ* 10.76 (s, 1H), 9.01 (s, 1H), 7.72–7.66 (m, 2H), 7.45 (t, *J* = 7.5 Hz, 1H), 7.34 (td, *J* = 7.7 Hz, *J* = 1.8 Hz, 1H), 7.23 (t, *J* = 7.5 Hz, 1H), 7.04–7.02 (m, 1H), 6.97–6.93 (m, 2H), 6.51 (s, 2H), 5.77 (dd, *J* = 12.0 Hz, *J* = 5.1 Hz, 1H), 4.64–4.54 (m, 2H), 3.94 (dd, *J* = 18.0 Hz, *J* = 12.0 Hz, 1H), 3.15 (dd, *J* = 18.0 Hz, *J* = 5.1 Hz, 1H); ^13^C NMR (100 MHz, DMSO-*d*_6_): *δ* 164.7, 155.3, 154.5, 151.8, 134.3, 133.0, 131.4, 131.4, 131.2, 128.6, 128.2, 126.0, 121.6, 121.2, 112.4, 66.5, 55.1, 44.5; HRMS (AP-ESI) *m*/*z* [M + H]^+^ calcd for C_18_H_18_BrN_4_O_4_: 433.0511, found: 433.0516.

Compounds 2b–2n were prepared in a similar manner as described for compound 2a.

### Preparation of 5-(2-(2-Hydrazinyl-2-oxoethoxy) phenyl)-3-(2-methoxyphenyl)-4,5-dihydro-1*H*-pyrazole-1-carboxamide (2o)

To a solution of compound 6d (0.77 g, 2 mmol) in hot methanol (10 mL) was added 80% hydrazine hydrate (0.5 g, 8 mmol). After stirred at 65 °C for 6 h, the solvent was evaporated and the residue was washed with water. The solid was dried under vacuum to give 0.36 g of compound 2o. White solid, yield: 47%, mp: 156.6–158.2 °C. ^1^H NMR (400 MHz, DMSO-*d*_6_): *δ* 9.26 (s, 1H), 7.91 (dd, *J* = 7.8 Hz, *J* = 1.8 Hz, 1H), 7.41–7.37 (m, 1H), 7.22–7.18 (m, 1H), 7.07 (d, *J* = 7.8 Hz, 1H), 7.00–6.90 (m, 4H), 6.48 (s, 2H), 5.71 (dd, *J* = 12.0 Hz, *J* = 5.0 Hz, 1H), 4.64–4.56 (m, 2H), 4.34 (s, 2H), 3.85 (dd, *J* = 18.5 Hz, *J* = 12.0 Hz, 1H), 3.34 (s, 3H), 3.11 (dd, *J* = 18.5 Hz, *J* = 5.0 Hz, 1H); ^13^C NMR (100 MHz, DMSO-*d*_6_): *δ* 167.0, 158.1, 155.4, 154.5, 151.2, 131.9, 131.6, 129.2, 128.4, 125.8, 121.5, 120.9, 112.7, 112.4, 66.9, 56.1, 54.7, 45.2; HRMS (AP-ESI) *m*/*z* [M + H]^+^ calcd for C_19_H_22_N_5_O_4_: 384.1672, found: 384.1663.

## Biological evaluation

### Enzymatic inhibition assay against APN *in vitro*

In the enzymatic inhibition assay against APN *in vitro*, l-Leu-*p*-nitroanilide was used as substrate and soluble APN from porcine kidney microsomes (Biocol) was used as enzyme. 50 mM PBS with pH 7.2 was used as buffer solution. Briefly, inhibitors (40 μL), PBS (145 μL), substrate (5 μL, 16 mmol L^−1^) and APN solution (10 μL, 0.15 IU mL^−1^) were added into 96-well plates. The mixture was incubated at 37 °C for 30 min. The optical density resulting from the hydrolysis product *p*-nitroanilide was measured at 405 nm with a plate reader (Varioskan, Thermo, USA).

### Anti-proliferation assay

The anti-proliferative activities of the selected compounds were evaluated using the MTT method. All the tumor cell lines were cultured in RPMI-1640 medium with 10% FBS at 37 °C in 5% CO_2_ humidified incubator. Firstly, cells (100 μL) were plated in 96-well plates and allowed to grow for 4 h. Then different concentrations of inhibitors (100 μL) were added. After incubation for 48 h, MTT solution (20 μL per well, 5 mg mL^−1^) was added and the mixture was incubated for additional 4 h. Subsequently, the medium were poured off and DMSO (200 μL) was added to dissolve the formed formazan. After shaking for 15 min, the optical density values were measured using a plate reader at 570 nm (Varioskan, Thermo, USA).

### *In vitro* anti-invasion assay

The carbonate membrane coated with matrigel in the upper chamber of transwell insert (BD BioCoat™ Matrigel™ Invasion Chambers) was rehydrated with RPMI-1640 culture medium (500 μL) containing 1% FBS for 2 h. After removing the medium, the upper and lower chambers were added the RPMI-1640 culture medium (100 μL) with 1% FBS containing the tested compounds and the RPMI-1640 culture medium (750 μL) with 10% FBS, respectively. Then, ES-2 cells suspending in the RPMI-1640 culture medium containing 1% FBS (400 μL, 2.5 × 10^5^ cells per mL) were added into the upper chambers. The system was placed at 37 °C in 5% CO_2_ for 8 h. The ES-2 cells could invade the matrigel and migrate from the upper surface of the carbonate membrane into the lower surface of that. ES-2 cells on the upper surface of the carbonate membrane were erased by cotton swabs. Then, ES-2 cells on the lower surface were fixed with methanol, stained with 0.1% crystal violet, of which the photographs were taken under an inverted microscope. The average numbers of ES-2 cells in five random fields (100×) per well were counted. The inhibition rate of ES-2 cells invasion was calculated as follows: (*A* − *B*)/*A* × 100%, *A* means the number of ES-2 cells in the control group, and *B* means the number of ES-2 cells in the treated group. The experiments were repeated three times.

### *In vivo* anti-metastatic assay

All animal procedures were performed in accordance with the Guidelines for Care and Use of Laboratory Animals of Shandong University and approved by the Animal Ethical and Welfare Committee (AEWC, China). The mice hepatoma H22 cell line was our laboratory-owned and 6 week old male Kunming mice were purchased from Center for New Drugs Evaluation of Shandong University, China. Firstly, H22 cells suspension (0.1 mL, 7.5 × 10^7^ mL^−1^) was injected *via* tail vein to establish a mice H22 pulmonary metastasis model. Subsequently, the mice were randomly divided into treatment and control groups. Treatment groups received 60 mg kg^−1^ d^−1^ of compound 2k or bestatin intraperitoneally for 12 days, while the control group received an equal volume of PBS intraperitoneally for the same period. The body weights were monitored every two days. Finally, the mice were sacrificed and the lungs were removed and fixed in Bouin's solution. After 24 h, the metastasis nodes on the surface of pulmonary lobes were counted.

## Statistical analysis

The statistical significance of differences between groups was assessed by Student's *t* test. *P* < 0.05 was taken as statistical significance.

## Conflicts of interest

There are no conflicts to declare.

## Supplementary Material

RA-011-D1RA03629G-s001
