# Supplementary material for: Development of pyrazoline-based derivatives as aminopeptidase N inhibitors to overcome cancer invasion and metastasis
Source: RSC Adv. 2021 Jun 17;11(35):21426–32. doi: 10.1039/d1ra03629g (PMC9034162; doi:10.1039/d1ra03629g)
Supplement: RA-011-D1RA03629G-s001 [file RA-011-D1RA03629G-s001.pdf]

Electronic Supplementary Material (ESI) for RSC Advances.
This journal is © The Royal Society of Chemistry 2021

Electronic Supplementary Information

S1. The spectra data of compounds 4b-4n, 5b-5n, 6b-6n and 2b-2n

(*E*)-1-(3-Bromophenyl)-3-(2-hydroxyphenyl)prop-2-en-1-one (4b)

Yellow solid, yield: 51%, mp: 142.2-144.2 °C. ^{1}H NMR (400 MHz, DMSO-d_6): δ 10.33 (s, 1H), 8.26 (s, 1H), 8.12-8.08 (m, 2H), 7.96-7.84 (m, 3H), 7.54 (t, J = 7.9 Hz, 1H), 7.30 (t, J = 8.5 Hz, 1H), 6.94 (d, J = 7.9 Hz, 1H), 6.89 (d, J = 7.9 Hz, 1H).

(*E*)-1-(4-Bromophenyl)-3-(2-hydroxyphenyl)prop-2-en-1-one (4c)

Yellow solid, yield: 55%, mp: 138.4-140.2 °C. ^{1}H NMR (400 MHz, DMSO-d_6): δ 10.32 (s, 1H), 8.09-8.04 (m, 3H), 7.89 (d, J = 8.5 Hz, 1H), 7.84 (d, J = 16.0 Hz, 1H), 7.78 (d, J = 8.5 Hz, 2H), 7.29 (t, J = 8.5 Hz, 1H), 6.94 (d, J = 8.5 Hz, 1H), 6.88 (t, J = 8.5 Hz, 1H).

(*E*)-3-(2-Hydroxyphenyl)-1-(2-methoxyphenyl)prop-2-en-1-one (4d)

Yellow solid, yield: 59%, mp: 108.4-110.2 °C. ^{1}H NMR (400 MHz, DMSO-d_6): δ 10.23 (s, 1H), 7.76 (d, J = 16.1 Hz, 1H), 7.64 (dd, J = 7.9 Hz, J = 1.7 Hz, 1H), 7.53 (t, J = 8.8 Hz, 1H), 7.47 (dd, J = 7.6 Hz, J = 1.8 Hz, 1H), 7.39 (d, J = 16.1 Hz, 1H), 7.26 (t, J = 8.6 Hz, 1H), 7.19 (d, J = 8.4 Hz, 1H), 7.06 (t, J = 7.4 Hz, 1H), 6.92 (dd, J = 8.2 Hz, J = 1.1 Hz, 1H), 6.85 (t, J = 8.6 Hz, 1H), 3.85 (s, 3H).

(*E*)-3-(2-Hydroxyphenyl)-1-(3-methoxyphenyl)prop-2-en-1-one (4e)

Yellow solid, yield: 60%, mp: 114.2-116.1 °C. ^{1}H NMR (400 MHz, DMSO-d_6): δ 10.30 (s, 1H), 8.06 (d, J = 15.7 Hz, 1H), 7.89 (d, J = 7.6 Hz, 1H), 7.83 (d, J = 15.7 Hz, 1H), 7.71 (d, J = 7.6 Hz, 1H), 7.57 (s, 1H), 7.49 (t, J = 7.6 Hz, 1H), 7.31-7.22 (m, 2H), 6.95 (d, J = 7.6 Hz, 1H), 6.88 (t, J = 7.6 Hz, 1H), 3.85 (s, 3H).

(*E*)-3-(2-Hydroxyphenyl)-1-(4-methoxyphenyl)prop-2-en-1-one (4f)

Yellow solid, yield: 54%, mp: 142.6-144.3 °C. ^{1}H NMR (400 MHz, DMSO-d_6): δ 10.25 (s, 1H), 8.13-8.10 (m, 2H), 8.02 (d, J = 15.7 Hz, 1H), 7.88-7.84 (m, 2H), 7.27 (t, J = 8.5 Hz, 1H), 7.10-7.07 (m, 2H), 6.94 (d, J = 8.5 Hz, 1H), 6.88 (d, J = 8.5 Hz, 1H), 3.87 (s, 3H).

(*E*)-3-(2-Hydroxyphenyl)-1-(*o*-tolyl)prop-2-en-1-one (4g)

Yellow solid, yield: 62%, mp: 134.4-136.2 °C. ^{1}H NMR (400 MHz, DMSO-d_6): δ 10.26 (s, 1H), 7.74-7.70 (m, 2H), 7.54 (d, J = 7.9 Hz, 1H), 7.43 (t, J = 7.5 Hz, 1H), 7.35-7.25 (m, 4H), 6.93 (d, J = 8.2 Hz, 1H), 6.86 (t, J = 7.5 Hz, 1H), 2.36 (s, 3H).

(*E*)-1-(2-Fluorophenyl)-3-(2-hydroxyphenyl)prop-2-en-1-one (4h)

Yellow solid, yield: 52%, mp: 154.2-156.2 °C. ^{1}H NMR (400 MHz, DMSO-d_6): δ 10.37 (s, 1H), 7.88 (d, J = 16.0 Hz, 1H), 7.76-7.62 (m, 3H), 7.47 (d, J = 16.0 Hz, 1H), 7.40-7.35 (m, 2H), 7.29 (t, J = 8.5 Hz, 1H), 6.95-6.93 (m, 1H), 6.87 (t, J = 7.5 Hz, 1H).

(*E*)-1-(2-Chlorophenyl)-3-(2-hydroxyphenyl)prop-2-en-1-one (4i)

Yellow solid, yield: 56%, mp: 134.5-136.2 °C. ^{1}H NMR (400 MHz, DMSO-d_6): δ 10.33 (s, 1H), 7.70 (d, J = 7.9 Hz, 1H), 7.64 (d, J = 16.3 Hz, 1H), 7.60-7.56 (m, 1H), 7.55-7.53 (m, 2H), 7.50-7.46 (m, 1H), 7.31-7.26 (m, 1H), 7.23 (d, J = 16.3 Hz, 1H), 6.92 (d, J = 8.2 Hz, 1H), 6.85 (t, J = 7.5 Hz, 1H).

(*E*)-1-(2,6-Difluorophenyl)-3-(2-hydroxyphenyl)prop-2-en-1-one (4j)

Yellow solid, yield: 59%, mp: 140.4-142.0 °C. ^{1}H NMR (400 MHz, DMSO-d_6): δ 10.41 (s, 1H), 7.72-7.68 (m, 2H), 7.66-7.60 (m, 1H), 7.33-7.22 (m, 4H), 6.93 (d, J = 8.2 Hz, 1H), 6.86 (t, J = 7.5 Hz, 1H).

(*E*)-1-(2,6-Dichlorophenyl)-3-(2-hydroxyphenyl)prop-2-en-1-one (4k)

Yellow solid, yield: 63%, mp: 220.4-222.2 °C. ^1H NMR (400 MHz, DMSO-d_6): δ 10.37 (s, 1H), 7.70 (dd, J = 7.9 Hz, J = 1.6 Hz, 1H), 7.63-7.61 (d, 2H), 7.56-7.49 (m, 2H), 7.30 (t, J = 8.6 Hz, 1H), 7.14 (d, J = 16.4 Hz, 1H), 6.91 (dd, J = 8.3 Hz, J = 1.1 Hz, 1H), 6.85 (t, J = 8.0 Hz, 1H).

(*E*)-1-(2,4-Dichlorophenyl)-3-(2-hydroxyphenyl)prop-2-en-1-one (4l)

Yellow solid, yield: 65%, mp: 144.6-146.5 °C. ^1H NMR (400 MHz, DMSO-d_6): δ 10.37 (s, 1H), 7.80-7.79 (m, 1H), 7.70 (dd, J = 8.0 Hz, 1H), 7.63 (d, J = 16.0 Hz, 1H), 7.59 (d, J = 1.6 Hz, 2H), 7.29 (t, J = 8.0 Hz, 1H), 7.24 (d, J = 16.0 Hz, 1H), 6.92 (d, J = 8.0 Hz, 1H), 6.86 (t, J = 8.0 Hz, 1H).

(*E*)-3-(2-Hydroxyphenyl)-1-(naphthalen-1-yl)prop-2-en-1-one (4m)

Yellow solid, yield: 48%, mp: 136.2-138.2 °C. ^1H NMR (400 MHz, DMSO-d_6): δ 10.28 (s, 1H), 8.26-8.23 (m, 1H), 8.14 (d, J = 8.2 Hz, 1H), 8.06-8.02 (m, 1H), 7.88 (d, J = 8.2 Hz, 1H), 7.84 (d, J = 16.1 Hz, 1H), 7.75 (dd, J = 7.9 Hz, J = 1.7 Hz, 1H), 7.66-7.58 (m, 3H), 7.50 (d, J = 16.1 Hz, 1H), 7.28 (t, J = 8.2 Hz, 1H), 6.92 (d, J = 8.2 Hz, 1H), 6.87 (t, J = 8.2 Hz, 1H).

(*E*)-3-(2-Hydroxyphenyl)-1-(naphthalen-2-yl)prop-2-en-1-one (4n)

Yellow solid, yield: 44%, 160.4-162.2 °C. ^1H NMR (400 MHz, DMSO-d_6): δ 10.33 (s, 1H), 8.89 (d, J = 1.7 Hz, 1H), 8.19-8.11 (m, 3H), 8.08-8.02 (m, 3H), 7.96 (dd, J = 7.9 Hz, J = 1.6 Hz, 1H), 7.71-7.63 (m, 2H), 7.31 (t, J = 8.6 Hz, 1H), 7.00-6.90 (m, 2H).

3-(3-Bromophenyl)-5-(2-hydroxyphenyl)-4,5-dihydro-1*H*-pyrazole-1-carboxamide (5b)

White solid, yield: 57%, mp: 258.4-260.2 °C. ^1H NMR (400 MHz, DMSO-d_6): δ 9.64 (s, 1H), 8.07 (t, J = 1.8 Hz, 1H), 7.71-7.68 (m, 1H), 7.60-7.57 (m, 1H), 7.37 (t, J = 7.9 Hz, 1H), 7.08-7.03 (m, 1H), 6.86-6.81 (m, 2H), 6.71 (td, J = 7.4 Hz, J = 1.2 Hz, 1H), 6.61 (s, 2H), 5.54 (dd, J = 12.0 Hz, J = 5.0 Hz, 1H), 3.74 (dd, J = 17.8 Hz, J = 12.0 Hz, 1H), 2.97 (dd, J = 17.8 Hz, J = 5.0 Hz, 1H).

3-(4-Bromophenyl)-5-(2-hydroxyphenyl)-4,5-dihydro-1*H*-pyrazole-1- carboxamide (5c)

White solid, yield: 54%, mp: 252.6-254.3 °C. ^1H NMR (400 MHz, DMSO-d_6): δ 9.66 (s, 1H), 7.74-7.72 (m, 2H), 7.62-7.60 (m, 2H), 7.06 (t, J = 7.7 Hz, 1H), 6.87-6.82 (m, 2H), 6.72 (t, J = 7.4 Hz, 1H), 6.56 (s, 2H), 5.55 (dd, J = 12.0 Hz, J = 5.0 Hz, 1H), 3.75 (dd, J = 17.7 Hz, J = 12.0 Hz, 1H), 2.96 (dd, J = 17.7 Hz, J = 5.0 Hz, 1H).

5-(2-Hydroxyphenyl)-3-(2-methoxyphenyl)-4,5-dihydro-1*H*-pyrazole-1-carboxamide (5d)

White solid, yield: 51%, mp: 200.4-202.3 °C. ^1H NMR (400 MHz, DMSO-d_6): δ 9.66 (s, 1H), 7.93 (dd, J = 7.8 Hz, J = 1.8 Hz, 1H), 7.38 (t, J = 8.7 Hz, 1H), 7.08-7.03 (m, 2H), 6.98 (t, J = 7.5 Hz, 1H), 6.83 (t, J = 7.5 Hz, 2H), 6.72 (t, J = 7.5 Hz, 1H), 6.48 (s, 2H), 5.49 (dd, J = 11.8 Hz, J = 4.5 Hz, 1H), 3.80 (dd, J = 18.3 Hz, J = 11.8 Hz, 1H), 3.77 (s, 3H), 3.03 (dd, J = 18.3 Hz, J = 4.5 Hz, 1H).

5-(2-Hydroxyphenyl)-3-(3-methoxyphenyl)-4,5-dihydro-1*H*-pyrazole-1-carboxamide (5e)

White solid, yield: 54%, mp: 206.2-208.2 °C. ^1H NMR (400 MHz, DMSO-d_6): δ 9.68 (s, 1H), 7.39 (s, 1H), 7.34-7.28 (m, 2H), 7.05 (td, J = 7.6 Hz, J = 1.7 Hz, 1H), 6.98-6.96 (m, 1H), 6.86-6.82 (m, 2H), 6.72 (t, J = 7.3 Hz, 1H), 6.58 (s, 2H), 5.54 (dd, J = 12.0 Hz, J = 4.8 Hz, 1H), 3.80 (s, 3H), 3.73 (dd, J = 17.7 Hz, J = 12.0 Hz, 1H), 2.96 (dd, J = 17.7 Hz, J = 4.8 Hz, 1H).

5-(2-Hydroxyphenyl)-3-(4-methoxyphenyl)-4,5-dihydro-1*H*-pyrazole-1-carboxamide (5f)

White solid, yield: 56%, mp: 230.4-232.2 °C. ^{1}H NMR (400 MHz, DMSO-d_6): δ 9.67 (s, 1H), 7.73-7.71 (m, 2H), 7.05 (t, J = 7.7 Hz, 1H), 6.98-6.96 (m, 2H), 6.84 (t, J = 7.9 Hz, 2H), 6.71 (t, J = 7.9 Hz, 1H), 6.48 (s, 2H), 5.52 (dd, J = 11.9 Hz, J = 4.6 Hz, 1H), 3.79 (s, 3H), 3.76-3.69 (m, 1H), 2.93 (dd, J = 17.6 Hz, J = 4.6 Hz, 1H).

5-(2-Hydroxyphenyl)-3-(*o*-tolyl)-4,5-dihydro-1*H*-pyrazole-1-carboxamide (5g)

White solid, yield: 55%, mp: 208.7-210.3 °C. ^{1}H NMR (400 MHz, DMSO-d_6): δ 9.68 (s, 1H), 7.43 (d, J = 7.5 Hz, 1H), 7.29-7.21 (m, 3H), 7.06 (t, J = 7.6 Hz, 1H), 6.90-6.83 (m, 2H), 6.73 (t, J = 7.4 Hz, 1H), 6.42 (s, 2H), 5.50 (dd, J = 11.8 Hz, J = 4.5 Hz, 1H), 3.86 (dd, J = 17.5 Hz, J = 11.8 Hz, 1H), 2.96 (dd, J = 17.5 Hz, J = 4.5 Hz, 1H), 2.58 (s, 3H).

3-(2-Fluorophenyl)-5-(2-hydroxyphenyl)-4,5-dihydro-1*H*-pyrazole-1-carboxamide (5h)

White solid, yield: 68%, mp: 242.3-244.3 °C. ^{1}H NMR (400 MHz, DMSO-d_6): δ 9.68 (s, 1H), 8.04 (t, J = 7.8 Hz, 1H), 7.48-7.43 (m, 1H), 7.26 (t, J = 9.0 Hz, 2H), 7.07 (t, J = 7.6 Hz, 1H), 6.88-6.83 (m, 2H), 6.73 (t, J = 7.4 Hz, 1H), 6.57 (s, 2H), 5.54 (dd, J = 12.2 Hz, J = 4.8 Hz, 1H), 3.85 (dd, J = 18.0 Hz, J = 12.2 Hz, 1H), 2.98 (dd, J = 18.0 Hz, J = 4.8 Hz, 1H).

3-(2-Chlorophenyl)-5-(2-hydroxyphenyl)-4,5-dihydro-1*H*-pyrazole-1-carboxamide (5i)

White solid, yield: 62%, mp: 216.5-218.2 °C. ^{1}H NMR (400 MHz, DMSO-d_6): δ 9.64 (s, 1H), 7.83 (d, J = 7.1 Hz, 1H), 7.51 (d, J = 7.5 Hz, 1H), 7.44-7.37 (m, 2H), 7.06 (t, J = 7.7 Hz, 1H), 6.89 (d, J = 7.7 Hz, 1H), 6.83 (d, J = 8.0 Hz, 1H), 6.74 (t, J = 7.4 Hz, 1H), 6.49 (s, 2H), 5.55 (dd, J = 12.0 Hz, J = 4.8 Hz, 1H), 3.91 (dd, J = 17.8 Hz, J = 12.0 Hz, 1H), 3.05 (dd, J = 17.8 Hz, J = 4.8 Hz, 1H).

3-(2,6-Difluorophenyl)-5-(2-hydroxyphenyl)-4,5-dihydro-1*H*-pyrazole-1-carboxamide (5j)

White solid, yield: 49%, mp: 204.4-206.2 °C. ^{1}H NMR (400 MHz, DMSO-d_6): δ 9.64 (s, 1H), 7.42-7.36 (m, 1H), 7.07 (td, J = 7.6 Hz, J = 1.7 Hz, 1H), 6.93-6.82 (m, 4H), 6.75 (d, J = 8.0 Hz, 1H), 6.40 (s, 2H), 5.55 (dd, J = 11.8 Hz, J = 4.0 Hz, 1H), 3.70 (dd, J = 17.8 Hz, J = 11.8 Hz, 1H), 2.90 (dd, J = 17.8 Hz, J = 4.0 Hz, 1H).

3-(2,6-Dichlorophenyl)-5-(2-hydroxyphenyl)-4,5-dihydro-1*H*-pyrazole-1-carboxamide (5k)

White solid, yield: 53%, mp: 244.5-246.2 °C. ^{1}H NMR (400 MHz, DMSO-d_6): δ 9.64 (s, 1H), 7.59-7.56 (m, 2H), 7.51-7.47 (m, 1H), 7.08 (td, J = 7.6 Hz, J = 1.6 Hz, 1H), 7.00 (dd, J = 7.6 Hz, J = 1.6 Hz, 1H), 6.84 (d, J = 8.0 Hz, 1H), 6.78 (d, J = 8.0 Hz, 1H), 6.50 (s, 2H), 5.64 (dd, J = 12.0 Hz, J = 5.2 Hz, 1H), 3.72 (dd, J = 18.0 Hz, J = 12.0 Hz, 1H), 2.76 (dd, J = 18.0 Hz, J = 5.2 Hz, 1H).

3-(2,4-Dichlorophenyl)-5-(2-hydroxyphenyl)-4,5-dihydro-1*H*-pyrazole-1-carboxamide (5l)

White solid, yield: 51%, mp: 228.4-230.2 °C. ^{1}H NMR (400 MHz, DMSO-d_6): δ 9.67 (s, 1H), 7.90 (d, J = 8.6 Hz, 1H), 7.68 (s, 1H), 7.48 (dd, J = 8.5 Hz, J = 2.2 Hz, 1H), 7.06 (td, J = 7.7 Hz, J = 1.7 Hz, 1H), 6.89-6.82 (m, 2H), 6.75-6.71 (m, 1H), 6.55 (s, 2H), 5.54 (dd, J = 12.0 Hz, J = 4.8 Hz, 1H), 3.91 (dd, J = 17.8 Hz, J = 12.0 Hz, 1H), 3.05 (dd, J = 17.8 Hz, J = 4.8 Hz, 1H).

5-(2-Hydroxyphenyl)-3-(naphthalen-1-yl)-4,5-dihydro-1*H*-pyrazole-1- carboxamide (5m)

White solid, yield: 56%, mp: 240.4-242.2 °C. ^{1}H NMR (400 MHz, DMSO-d_6): δ 9.67 (s, 1H), 9.27-9.24 (m, 1H), 7.98 (dt, J = 8.2 Hz, J = 1.6 Hz, 2H), 7.70-7.64 (m, 2H), 7.61-7.57 (m, 1H),

7.53-7.50 (m, 1H), 7.06 (td, J = 7.7 Hz, J = 1.6 Hz, 1H), 6.95 (dd, J = 7.7 Hz, J = 1.6 Hz, 1H), 6.85 (dd, J = 8.0 Hz, J = 1.1 Hz, 1H), 6.74 (td, J = 7.4 Hz, J = 1.1 Hz, 1H), 6.56 (s, 2H), 5.56 (dd, J = 11.9 Hz, J = 4.6 Hz, 1H), 4.04 (dd, J = 17.3 Hz, J = 11.9 Hz, 1H), 3.13 (dd, J = 17.3 Hz, J = 4.6 Hz, 1H).

5-(2-Hydroxyphenyl)-3-(naphthalen-2-yl)-4,5-dihydro-1*H*-pyrazole-1-carboxamide (5n)

White solid, yield: 52%, mp: 272.2-274.2 °C. ^{1}H NMR (400 MHz, DMSO-d_6): δ 9.69 (s, 1H), 8.17-8.09 (m, 2H), 7.93 (d, J = 8.7 Hz, 3H), 7.56-7.52 (m, 2H), 7.06 (t, J = 7.7 Hz, 1H), 6.89-6.84 (m, 2H), 6.72 (t, J = 7.4 Hz, 1H), 6.60 (s, 2H), 5.61 (dd, J = 12.0 Hz, J = 4.8 Hz, 1H), 3.87 (dd, J = 17.6 Hz, J = 12.0 Hz, 1H), 3.12 (dd, J = 17.6 Hz, J = 4.8 Hz, 1H).

Methyl 2-(2-(3-(3-bromophenyl)-1-carbamoyl-4,5-dihydro-1*H*-pyrazol-5-yl) phenoxy)acetate (6b)

White solid, yield: 61%, mp: 188.4-190.2 °C. ^{1}H NMR (400 MHz, DMSO-d_6): δ 8.04 (s, 1H), 7.66 (dt, J = 7.8 Hz, J = 1.3 Hz, 1H), 7.60-7.58 (m, 1H), 7.37 (t, J = 7.8 Hz, 1H), 7.23-7.18 (m, 1H), 6.97-6.89 (m, 3H), 6.66 (s, 2H), 5.62 (dd, J = 12.0 Hz, J = 5.0 Hz, 1H), 4.88 (s, 2H), 3.77 (dd, J = 17.8 Hz, J = 12.0 Hz, 1H), 3.70 (s, 3H), 3.03 (dd, J = 17.8 Hz, J = 5.0 Hz, 1H).

Methyl 2-(2-(3-(4-bromophenyl)-1-carbamoyl-4,5-dihydro-1*H*-pyrazol-5-yl) phenoxy)acetate (6c)

White solid, yield: 59%, mp: 190.3-192.2 °C. ^{1}H NMR (400 MHz, DMSO-d_6): δ 7.69 (d, J = 8.7 Hz, 2H), 7.62 (d, J = 8.7 Hz, 2H), 7.22-7.18 (m, 1H), 6.97-6.89 (m, 3H), 6.60 (s, 2H), 5.62 (dd, J = 12.0 Hz, J = 5.0 Hz, 1H), 4.88 (s, 2H), 3.78 (dd, J = 17.8 Hz, J = 12.0 Hz, 1H), 3.70 (s, 3H), 3.02 (dd, J = 17.8 Hz, J = 5.0 Hz, 1H).

Methyl 2-(2-(1-carbamoyl-3-(2-methoxyphenyl)-4,5-dihydro-1*H*-pyrazol-5-yl)phenoxy) acetate (6d)

White solid, yield: 67%, mp: 176.4-178.3 °C. ^{1}H NMR (400 MHz, DMSO-d_6): δ 7.92 (d, J = 7.8 Hz, 1H), 7.38 (t, J = 8.7 Hz, 1H), 7.21-7.17 (m, 1H), 7.06 (d, J = 8.4 Hz, 1H), 7.00-6.91 (m, 4H), 6.50 (s, 2H), 5.57 (dd, J = 11.8 Hz, J = 4.6 Hz, 1H), 4.96-4.85 (m, 2H), 3.81 (dd, J = 18.4 Hz, J = 11.8 Hz, 1H), 3.76 (s, 3H), 3.70 (s, 3H), 3.11 (dd, J = 18.4 Hz, J = 4.6 Hz, 1H).

Methyl 2-(2-(1-carbamoyl-3-(3-methoxyphenyl)-4,5-dihydro-1*H*-pyrazol-5-yl)phenoxy) acetate (6e)

White solid, yield: 61%, mp: 148.8-150.5 °C. ^{1}H NMR (400 MHz, DMSO-d_6): δ 7.37-7.18 (m, 4H), 6.99-6.89 (m, 4H), 6.61 (s, 2H), 5.62 (dd, J = 12.0 Hz, J = 4.9 Hz, 1H), 4.89 (s, 2H), 3.79-3.71 (m, 7H), 3.03 (dd, J = 17.7 Hz, J = 4.9 Hz, 1H).

Methyl 2-(2-(1-carbamoyl-3-(4-methoxyphenyl)-4,5-dihydro-1*H*-pyrazol-5-yl)phenoxy) acetate (6f)

White solid, yield: 66%, mp: 160.6-162.5 °C. ^{1}H NMR (400 MHz, DMSO-d_6): δ 7.69-7.66 (m, 2H), 7.22-7.17 (m, 1H), 6.99-6.89 (m, 5H), 6.50 (s, 2H), 5.60 (dd, J = 11.8 Hz, J = 4.8 Hz, 1H), 4.89 (s, 2H), 3.78-3.71 (m, 7H), 2.99 (dd, J = 17.7 Hz, J = 4.8 Hz, 1H).

Methyl 2-(2-(1-carbamoyl-3-(*o*-tolyl)-4,5-dihydro-1*H*-pyrazol-5-yl)phenoxy) acetate (6g)

White solid, yield: 58%, mp: 134.6-136.4 °C. ^{1}H NMR (400 MHz, DMSO-d_6): δ 7.39 (d, J = 7.5 Hz, 1H), 7.28 (d, J = 4.3 Hz, 2H), 7.29-7.28 (m, 1H), 7.26-7.18 (m, 1H), 6.96-6.91 (m, 3H), 6.44 (s, 2H), 5.57 (dd, J = 11.8 Hz, J = 4.6 Hz, 1H), 4.89 (s, 2H), 3.88 (dd, J = 17.6 Hz, J = 11.8 Hz, 1H), 3.69 (s, 3H), 3.04 (dd, J = 17.6 Hz, J = 4.6 Hz, 1H), 2.57 (s, 3H).

Methyl 2-(2-(1-carbamoyl-3-(2-fluorophenyl)-4,5-dihydro-1*H*-pyrazol-5-yl)

phenoxy)acetate (6h)

White solid, yield: 54%, mp: 132.4-134.1 °C. ^{1}H NMR (400 MHz, DMSO-d_6): δ 8.04 (t, J = 7.8 Hz, 1H), 7.48-7.43 (m, 1H), 7.28-7.19 (m, 3H), 6.96-6.90 (m, 3H), 6.60 (s, 2H), 5.61 (dd, J = 12.0 Hz, J = 5.0 Hz, 1H), 4.94-4.81 (m, 2H), 3.86 (dd, J = 18.2 Hz, J = 12.0 Hz, 1H), 3.69 (s, 3H), 3.11-3.04 (m, 1H).

Methyl 2-(2-(1-carbamoyl-3-(2-chlorophenyl)-4,5-dihydro-1*H*-pyrazol-5-yl) phenoxy)acetate (6i)

White solid, yield: 57%, mp: 148.4-150.3 °C. ^{1}H NMR (400 MHz, DMSO-d_6): δ 7.81 (d, J = 6.9 Hz, 1H), 7.52-7.50 (m, 1H), 7.44-7.37 (m, 2H), 7.23-7.19 (m, 1H), 6.98-6.91 (m, 3H), 6.54 (s, 2H), 5.63 (dd, J = 11.9 Hz, J = 4.8 Hz, 1H), 4.94-4.85 (m, 2H), 3.94 (dd, J = 17.9 Hz, J = 11.9 Hz, 1H), 3.68 (s, 3H), 3.13 (dd, J = 17.9 Hz, J = 4.8 Hz, 1H).

Methyl 2-(2-(1-carbamoyl-3-(2,6-difluorophenyl)-4,5-dihydro-1*H*-pyrazol-5-yl)phenoxy)acetate (6j)

White solid, yield: 51%, mp: 136.5-138.2 °C. ^{1}H NMR (400 MHz, DMSO-d_6): δ 7.42-7.36 (m, 1H), 7.23-7.19 (m, 1H), 6.98-6.84 (m, 5H), 6.41 (s, 2H), 5.61 (dd, J = 11.7 Hz, J = 4.2 Hz, 1H), 4.91-4.84 (m, 2H), 3.79-3.65 (m, 1H), 3.69 (s, 3H), 2.93 (dd, J = 17.8 Hz, J = 4.2 Hz, 1H).

Methyl 2-(2-(1-carbamoyl-3-(2,6-dichlorophenyl)-4,5-dihydro-1*H*-pyrazol-5-yl)phenoxy)acetate (6k)

White solid, yield: 54%, mp: 196.4-198.2 °C. ^{1}H NMR (400 MHz, DMSO-d_6): δ 7.59-7.56 (m, 2H), 7.51-7.47 (m, 1H), 7.25-7.21 (m, 1H), 7.09-7.06 (m, 1H), 6.99-6.95 (m, 2H), 6.54 (s, 2H), 5.71 (dd, J = 12.0 Hz, J = 5.4 Hz, 1H), 4.94-4.85 (m, 2H), 3.76 (dd, J = 18.2 Hz, J = 12.0 Hz, 1H), 3.69 (s, 3H), 2.83 (dd, J = 18.2 Hz, J = 5.4 Hz, 1H).

Methyl 2-(2-(1-carbamoyl-3-(2,4-dichlorophenyl)-4,5-dihydro-1*H*-pyrazol-5-yl)phenoxy)acetate (6l)

White solid, yield: 58%, mp: 196.4-198.3 °C. ^{1}H NMR (400 MHz, DMSO-d_6): δ 7.87 (d, J = 8.6 Hz, 1H), 7.68 (d, J = 2.2 Hz, 1H), 7.49 (dd, J = 8.6 Hz, J = 2.2 Hz, 1H), 7.21 (t, J = 8.9 Hz, 1H), 6.98-6.91 (m, 3H), 6.57 (s, 2H), 5.62 (dd, J = 12.0 Hz, J = 5.0 Hz, 1H), 4.89 (s, 2H), 3.93 (dd, J = 17.8 Hz, J = 12.0 Hz, 1H), 3.68 (s, 3H), 3.14 (dd, J = 17.8 Hz, J = 5.0 Hz, 1H).

Methyl 2-(2-(1-carbamoyl-3-(naphthalen-1-yl)-4,5-dihydro-1*H*-pyrazol-5-yl) phenoxy)acetate (6m)

White solid, yield: 62%, mp: 206.2-208.2 °C. ^{1}H NMR (400 MHz, DMSO-d_6): δ 9.25-9.23 (m, 1H), 7.98 (dd, J = 8.0 Hz, J = 1.9 Hz, 2H), 7.69-7.64 (m, 2H), 7.62-7.58 (m, 1H), 7.54-7.51 (m, 1H), 7.24-7.19 (m, 1H), 7.04 (dd, J = 7.6 Hz, J = 1.7 Hz, 1H), 6.98-6.92 (m, 2H), 6.60 (s, 2H), 5.65 (dd, J = 11.9 Hz, J = 4.6 Hz, 1H), 4.90 (s, 2H), 4.07 (dd, J = 17.4 Hz, J = 11.9 Hz, 1H), 3.69 (s, 3H), 3.21 (dd, J = 17.4 Hz, J = 4.6 Hz, 1H).

Methyl2-(2-(1-carbamoyl-3-(naphthalen-2-yl)-4,5-dihydro-1*H*-pyrazol-5-yl) phenoxy)acetate (6n)

White solid, yield: 65%, mp: 136.2-138.2 °C. ^{1}H NMR (400 MHz, DMSO-d_6): δ 8.15 (d, J = 8.8 Hz, 1H), 8.04 (d, J = 1.6 Hz, 1H), 7.95-7.92 (m, 3H), 7.56-7.52 (m, 2H), 7.21 (t, J = 8.6 Hz, 1H), 6.98-6.90 (m, 3H), 6.64 (s, 2H), 5.69 (dd, J = 11.9 Hz, J = 4.8 Hz, 1H), 4.91 (s, 2H), 3.90 (dd, J = 17.6 Hz, J = 11.9 Hz, 1H), 3.72 (s, 3H), 3.16 (dd, J = 17.6 Hz, J = 4.8 Hz, 1H).

3-(3-Bromophenyl)-5-(2-(2-(hydroxyamino)-2-oxoethoxy)phenyl)-4,5-dihydro-1*H*-pyrazole-1-carboxamide (2b)

White solid, yield: 55%, mp: 164.4-166.4 °C. ^{1}H NMR (400 MHz, DMSO-d_6): δ 10.77 (s, 1H),

9.03 (s, 1H), 8.06 (s, 1H), 7.71 (d, J = 7.8 Hz, 1H), 7.60 (d, J = 8.0 Hz, 1H), 7.39 (t, J = 7.9 Hz, 1H), 7.25-7.19 (m, 1H), 6.99-6.85 (m, 3H), 6.69 (s, 2H), 5.78 (dd, J = 12.0 Hz, J = 5.0 Hz, 1H), 4.66-4.56 (m, 2H), 3.76 (dd, J = 18.0 Hz, J = 12.0 Hz, 1H), 3.13 (dd, J = 18.0 Hz, J = 5.0 Hz, 1H); ^{13}C NMR (100 MHz, DMSO-d_6): δ 164.8, 155.3, 154.5, 150.4, 134.5, 132.6, 131.4, 131.2, 129.1, 128.6, 125.9, 125.8, 122.5, 121.6, 112.4, 66.6, 55.2, 41.4; HRMS (AP-ESI) m/z [M+H]$^+$ calcd for $C_{18}H_{18}BrN_4O_4$: 433.0511, found: 433.0508.

3-(4-Bromophenyl)-5-(2-(2-(hydroxyamino)-2-oxoethoxy)phenyl)-4,5-dihydro-1*H*-pyrazole-1-carboxamide (2c)

White solid, yield: 56%, mp: 190.2-192.2 °C. ^{1}H NMR (400 MHz, DMSO-d_6): δ 10.76 (s, 1H), 9.02 (s, 1H), 7.72 (d, J = 8.4 Hz, 2H), 7.64 (d, J = 8.4 Hz, 2H), 7.25-7.20 (m, 1H), 6.98-6.90 (m, 3H), 6.63 (s, 2H), 5.77 (dd, J = 12.0 Hz, J = 5.1 Hz, 1H), 4.66-4.53 (m, 2H), 3.77 (dd, J = 17.9 Hz, J = 12.0 Hz, 1H), 3.11 (dd, J = 17.9 Hz, J = 5.1 Hz, 1H); ^{13}C NMR (100 MHz, DMSO-d_6): δ 164.7, 155.3, 154.5, 150.9, 132.0, 131.4, 131.3, 128.7, 128.6, 125.8, 123.3, 121.6, 112.4, 66.5, 55.2, 41.5; HRMS (AP-ESI) m/z [M+H]$^+$ calcd for $C_{18}H_{18}BrN_4O_4$: 433.0511, found: 433.0510.

5-(2-(2-(Hydroxyamino)-2-oxoethoxy)phenyl)-3-(2-methoxyphenyl)-4,5-dihydro-1*H*-pyrazole-1-carboxamide (2d)

White solid, yield: 51%, mp: 198.4-200.2 °C. ^{1}H NMR (400 MHz, DMSO-d_6): δ 10.78 (s, 1H), 9.02 (s, 1H), 7.92 (dd, J = 7.8 Hz, J = 1.8 Hz, 1H), 7.41-7.37 (m, 1H), 7.23-7.19 (m, 1H), 7.07 (d, J = 8.3 Hz, 1H), 7.01-6.89 (m, 4H), 6.52 (s, 2H), 5.72 (dd, J = 11.9 Hz, J = 5.0 Hz, 1H), 4.64-4.55 (m, 2H), 3.85 (dd, J = 18.5 Hz, J = 11.9 Hz, 1H), 3.78 (s, 3H), 3.11 (dd, J = 18.5 Hz, J = 5.0 Hz, 1H); ^{13}C NMR (100 MHz, DMSO-d_6): δ 164.8, 158.1, 155.4, 154.4, 151.2, 131.9, 131.6, 129.2, 128.5, 125.7, 121.6, 120.9, 120.9, 112.7, 112.3, 66.4, 56.1, 54.5, 45.2; HRMS (AP-ESI) m/z [M+H]$^+$ calcd for $C_{19}H_{21}N_4O_5$: 385.1512, found: 385.1512.

5-(2-(2-(Hydroxyamino)-2-oxoethoxy)phenyl)-3-(3-methoxyphenyl)-4,5-dihydro-1*H*-pyrazole-1-carboxamide (2e)

White solid, yield: 55%, mp: 208.5-210.3 °C. ^{1}H NMR (400 MHz, DMSO-d_6): δ 10.77 (s, 1H), 9.02 (s, 1H), 7.41-7.20 (m, 4H), 7.00-6.87 (m, 4H), 6.63 (s, 2H), 5.76 (dd, J = 12.0 Hz, J = 5.0 Hz, 1H), 4.66-4.56 (m, 2H), 3.80 (s, 3H), 3.77-3.73 (m, 1H), 3.11 (dd, J = 18.0 Hz, J = 5.0 Hz, 1H); ^{13}C NMR (100 MHz, DMSO-d_6): δ 164.8, 159.8, 155.3, 154.5, 151.7, 133.4, 131.6, 130.1, 128.6, 125.8, 121.6, 119.4, 116.2, 112.4, 111.5, 66.6, 55.6, 54.9, 41.7; HRMS (AP-ESI) m/z [M+H]$^+$ calcd for $C_{19}H_{21}N_4O_5$: 385.1512, found: 385.1511.

5-(2-(2-(Hydroxyamino)-2-oxoethoxy)phenyl)-3-(4-methoxyphenyl)-4,5-dihydro-1*H*-pyrazole-1-carboxamide (2f)

White solid, yield: 58%, mp: 168.4-170.2 °C. ^{1}H NMR (400 MHz, DMSO-d_6): δ 10.77 (s, 1H), 9.02 (s, 1H), 7.71 (d, J = 8.7 Hz, 2H), 7.23-7.19 (m, 1H), 6.99-6.95 (m, 3H), 6.93-6.89 (m, 2H), 6.52 (s, 2H), 5.73 (dd, J = 12.0 Hz, J = 4.8 Hz, 1H), 4.65-4.55 (m, 2H), 3.79 (s, 3H), 3.76-3.71 (m, 1H), 3.08 (dd, J = 17.8 Hz, J = 4.8 Hz, 1H); ^{13}C NMR (100 MHz, DMSO-d_6): δ 164.8, 160.9, 155.4, 154.5, 151.7, 131.7, 128.5, 128.4, 125.8, 124.6, 121.6, 114.4, 112.3, 66.5, 55.7, 54.7, 41.9; HRMS (AP-ESI) m/z [M+H]$^+$ calcd for $C_{19}H_{21}N_4O_5$: 385.1512, found: 385.1543.

5-(2-(2-(Hydroxyamino)-2-oxoethoxy)phenyl)-3-(*o*-tolyl)-4,5-dihydro-1*H*- pyrazole-1-carboxamide (2g)

White solid, yield: 55%, mp: 144.5-146.2 °C. ^{1}H NMR (400 MHz, DMSO-d_6): δ 10.62 (s, 1H), 9.26 (s, 1H), 7.44 (d, J = 7.5 Hz, 1H), 7.29-7.28 (m, 2H), 7.26-7.19 (m, 2H), 6.99-6.87 (m, 3H), 6.45 (s, 2H), 5.70 (dd, J = 11.8 Hz, J = 4.8 Hz, 1H), 4.64-4.54 (m, 2H), 3.87 (dd, J = 17.7 Hz, J =

11.8 Hz, 1H), 3.12 (dd, *J* = 17.7 Hz, *J* = 4.8 Hz, 1H), 2.58 (s, 3H); ^{13}C NMR (100 MHz, DMSO-d_6): δ 155.4, 154.6, 152.9, 137.5, 131.8, 131.5, 130.8, 129.3, 128.5, 126.3, 125.7, 121.5, 112.5, 66.6, 54.1, 44.1, 23.3; HRMS (AP-ESI) m/z [M+H]$^+$ calcd for $C_{19}H_{21}N_4O_4$: 369.1563, found: 369.1556.

3-(2-Fluorophenyl)-5-(2-(2-(hydroxyamino)-2-oxoethoxy)phenyl)-4,5-dihydro-1*H*-pyrazole-1-carboxamide (2h)

White solid, yield: 59%, mp: 124.4-126.1 °C. ^{1}H NMR (400 MHz, DMSO-d_6): δ 10.77 (s,1H), 9.05 (s, 1H), 8.03 (t, *J* = 7.8 Hz, 1H), 7.49-7.43 (m, 1H), 7.29-7.20 (m, 3H), 6.99-6.90 (m, 3H), 6.60 (s, 2H), 5.76 (dd, *J* = 12.0 Hz, *J* = 5.1 Hz, 1H), 4.63-4.54 (m, 2H), 3.88 (dd, *J* = 18.3 Hz, *J* = 12.0 Hz, 1H), 3.12-3.05 (m, 1H); ^{13}C NMR (100 MHz, DMSO-d_6): δ 160.5 (d, *J* = 250.0 Hz), 155.3, 154.5, 148.0, 132.0 (d, *J* = 8.0 Hz), 131.4, 129.4, 128.6, 125.7, 125.0, 121.5, 120.0, 119.9, 116.9 (d, *J* = 22.0 Hz), 112.4, 66.5, 54.9, 44.0; HRMS (AP-ESI) m/z [M+H]$^+$ calcd for $C_{18}H_{18}FN_4O_4$: 373.1312, found: 373.1304.

3-(2-Chlorophenyl)-5-(2-(2-(hydroxyamino)-2-oxoethoxy)phenyl)-4,5-dihydro-1*H*-pyrazole-1-carboxamide (2i)

White solid, yield: 59%, mp: 172.2-174.1 °C. ^{1}H NMR (400 MHz, DMSO-d_6): δ 10.75 (s, 1H), 9.00 (s, 1H), 7.81 (dd, *J* = 7.1 Hz, *J* = 2.4 Hz, 1H), 7.52 (dd, *J* = 7.6 Hz, *J* = 1.8 Hz, 1H), 7.45-7.38 (m, 2H), 7.22 (t, *J* = 7.1 Hz, 1H), 7.01-6.93 (m, 3H), 6.54 (s, 2H), 5.77 (dd, *J* = 12.0 Hz, *J* = 5.0 Hz, 1H), 4.63-4.53 (m, 2H), 3.94 (dd, *J* = 18.0 Hz, *J* = 12.0 Hz, 1H), 3.17 (dd, *J* = 18.0 Hz, *J* = 5.0 Hz, 1H); ^{13}C NMR (100 MHz, DMSO-d_6): δ 164.7, 155.3, 154.5, 150.8, 132.0, 131.2, 131.1, 131.0, 128.6, 127.7, 125.8, 121.6, 112.4, 66.5, 55.2, 44.5; HRMS (AP-ESI) m/z [M+H]$^+$ calcd for $C_{18}H_{18}ClN_4O_4$: 389.1017, found: 389.1015.

3-(2,6-Difluorophenyl)-5-(2-(2-(hydroxyamino)-2-oxoethoxy)phenyl)-4,5-dihydro-1*H*-pyrazole-1-carboxamide (2j)

White solid, yield: 45%, mp: 186.2-188.2 °C. ^{1}H NMR (400 MHz, DMSO-d_6): δ 10.75 (s, 1H), 8.97 (s, 1H), 7.42-7.37 (m, 1H), 7.25-7.21 (m, 1H), 7.05-7.03 (m, 1H), 6.97-6.84 (m, 4H), 6.42 (s, 2H), 5.77 (dd, *J* = 12.0 Hz, *J* = 4.4 Hz, 1H), 4.63-4.53 (m, 2H), 3.74 (dd, *J* = 18.0 Hz, *J* = 12.0 Hz, 1H), 2.97 (dd, *J* = 18.0 Hz, *J* = 4.4 Hz, 1H); ^{13}C NMR (100 MHz, DMSO-d_6): δ 160.2 (d, *J* = 292 Hz), 160.1 (d, *J* = 298 Hz), 155.1, 154.3, 146.6, 131.8, 131.7, 131.5, 128.6, 125.9, 121.6, 112.2, 108.8, 108.4, 108.2, 66.4, 53.9, 45.4; HRMS (AP-ESI) m/z [M+H]$^+$ calcd for $C_{18}H_{17}F_2N_4O_4$: 391.1218, found: 391.1211.

3-(2,6-Dichlorophenyl)-5-(2-(2-(hydroxyamino)-2-oxoethoxy)phenyl)-4,5-dihydro-1*H*-pyrazole-1-carboxamide (2k)

White solid, yield: 48%, mp: 200.2-202.2 °C. ^{1}H NMR (400 MHz, DMSO-d_6): δ 10.74 (s, 1H), 8.98 (s, 1H), 7.59-7.56 (m, 2H), 7.52-7.48 (m, 1H), 7.26-7.22 (m, 1H), 7.12-7.10 (m, 1H), 6.98 (t, *J* = 8.2 Hz, 2H), 6.53 (s, 2H), 5.86 (dd, *J* = 12.2 Hz, *J* = 5.4 Hz, 1H), 4.63-4.53 (m, 2H), 3.79 (dd, *J* = 18.4 Hz, *J* = 12.2 Hz, 1H), 2.84 (dd, *J* = 18.4 Hz, *J* = 5.4 Hz, 1H); ^{13}C NMR (100 MHz, DMSO-d_6): δ 164.7, 155.2, 154.3, 149.4, 134.5, 132.4, 131.5, 131.0, 128.9, 128.6, 125.9, 121.6, 112.3, 66.6, 54.7, 44.9; HRMS (AP-ESI) m/z [M+H]$^+$ calcd for $C_{18}H_{17}Cl_2N_4O_4$: 423.0627, found: 423.0623.

3-(2,4-Dichlorophenyl)-5-(2-(2-(hydroxyamino)-2-oxoethoxy)phenyl)-4,5-dihydro-1*H*-pyrazole-1-carboxamide (2l)

White solid, yield: 51%, mp: 196.3-198.2 °C. ^{1}H NMR (400 MHz, DMSO-d_6): δ 10.76 (s, 1H), 9.02 (s, 1H), 7.87 (d, *J* = 8.5 Hz, 1H), 7.68 (s, 1H), 7.49 (dd, *J* = 8.5 Hz, *J* = 2.2 Hz, 1H), 7.25-

7.21 (m, 1H), 7.00-6.92 (m, 3H), 6.59 (s, 2H), 5.77 (dd, J = 12.0 Hz, J = 5.2 Hz, 1H), 4.64-4.54 (m, 2H), 3.94 (dd, J = 18.0 Hz, J = 12.0 Hz, 1H), 3.18 (dd, J = 18.0 Hz, J = 5.2 Hz, 1H); ^{13}C NMR (100 MHz, DMSO-d_6): δ 164.7, 155.2, 154.5, 149.7, 134.8, 132.9, 132.3, 131.1, 130.5, 129.9, 128.7, 127.9, 125.9, 121.6, 112.4, 66.4, 55.4, 44.3; HRMS (AP-ESI) m/z $[M+H]^+$ calcd for $C_{18}H_{17}Cl_2N_4O_4$: 423.0627, found: 423.0621.

5-(2-(2-(Hydroxyamino)-2-oxoethoxy)phenyl)-3-(naphthalen-1-yl)-4,5-dihydro-1*H*-pyrazole-1-carboxamide (2m)

White solid, yield: 53%, mp: 204.2-206.2 °C. ^1H NMR (400 MHz, DMSO-d_6): δ 10.80 (s, 1H), 9.29 (d, J = 8.7 Hz, 1H), 9.03 (s, 1H), 7.98 (d, J = 8.1 Hz, 2H), 7.70-7.66 (m, 2H), 7.62-7.58 (m, 1H), 7.55-7.51 (m, 1H), 7.25-7.21 (m, 1H), 7.05-7.03 (m, 1H), 7.00-6.92 (m, 2H), 6.65 (s, 2H), 5.78 (dd, J = 11.9 Hz, J = 4.8 Hz, 1H), 4.68-4.57 (m, 2H), 4.05 (dd, J = 17.5 Hz, J = 11.9 Hz, 1H), 3.33-3.28 (m, 1H); ^{13}C NMR (100 MHz, DMSO-d_6): δ 164.8, 155.4, 154.7, 152.8, 134.0, 131.5, 130.8, 130.3, 129.0, 128.9, 128.6, 128.2, 128.2, 127.5, 126.6, 125.8, 125.5, 121.6, 112.4, 66.5, 53.8, 44.3; HRMS (AP-ESI) m/z $[M+H]^+$ calcd for $C_{22}H_{21}N_4O_4$: 405.1563, found: 405.1562.

5-(2-(2-(Hydroxyamino)-2-oxoethoxy)phenyl)-3-(naphthalen-2-yl)-4,5-dihydro-1*H*-pyrazole-1-carboxamide (2n)

White solid, yield: 51%, mp: 166.2-168.2 °C. ^1H NMR (400 MHz, DMSO-d_6): δ 10.31 (s, 1H), 9.48 (s, 1H), 8.20 (d, J = 8.9 Hz, 1H), 8.12 (d, J = 6.5 Hz, 1H), 7.99-7.96 (m, 3H), 7.60-7.54 (m, 2H), 7.28-7.24 (m, 1H), 7.05-6.89 (m, 3H), 6.68 (s, 2H), 5.88 (dd, J = 12.0 Hz, J = 5.0 Hz, 1H), 4.72-4.61 (m, 2H), 3.94 (dd, J = 17.7 Hz, J = 12.0 Hz, 1H), 3.26 (dd, J = 17.7 Hz, J = 5.0 Hz, 1H); ^{13}C NMR (100 MHz, DMSO-d_6): δ 164.7, 155.3, 154.5, 151.9, 133.8, 133.2, 131.6, 129.7, 128.7, 128.6, 128.4, 128.1, 127.4, 127.1, 127.1, 125.7, 123.8, 121.6, 112.5, 66.7, 55.1, 41.7; HRMS (AP-ESI) m/z $[M+H]^+$ calcd for $C_{22}H_{21}N_4O_4$: 405.1563, found: 405.1558.

S2. The IC_{50} graphs of compounds against APN

A

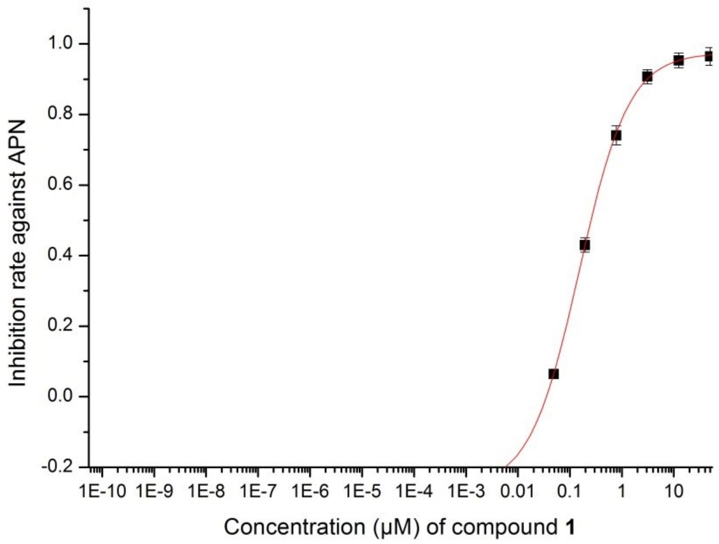

B

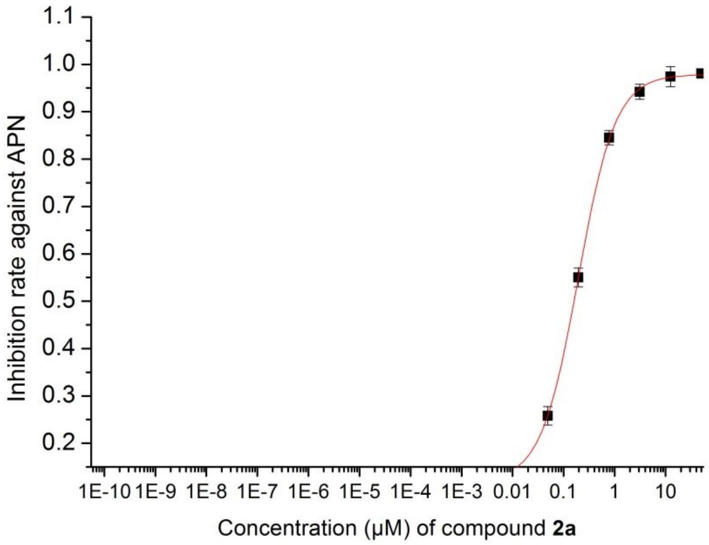
Inhibition rate against APN
1.1
1.0
0.9
0.8
0.7
0.6
0.5
0.4
0.3
0.2
1E-10 1E-9 1E-8 1E-7 1E-6 1E-5 1E-4 1E-3 0.01 0.1 1 10
Concentration (μM) of compound **2a**

C

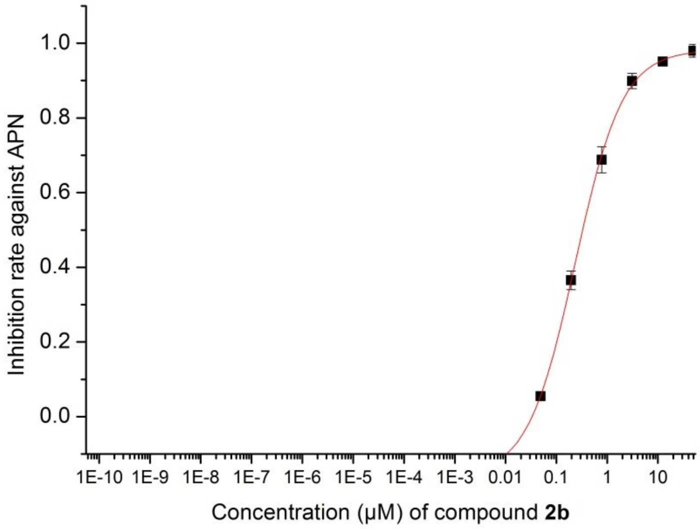
Inhibition rate against APN
1.0
0.8
0.6
0.4
0.2
0.0
1E-10 1E-9 1E-8 1E-7 1E-6 1E-5 1E-4 1E-3 0.01 0.1 1 10
Concentration (μM) of compound **2b**

D

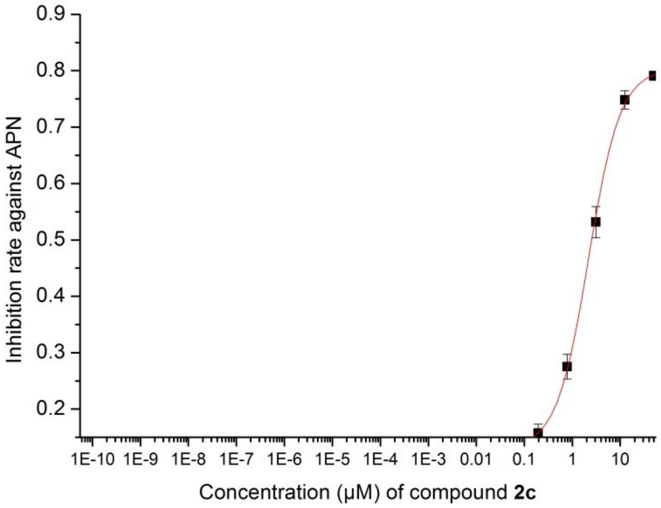
Inhibition rate against APN
0.9
0.8
0.7
0.6
0.5
0.4
0.3
0.2
1E-10 1E-9 1E-8 1E-7 1E-6 1E-5 1E-4 1E-3 0.01 0.1 1 10
Concentration (μM) of compound **2c**

E

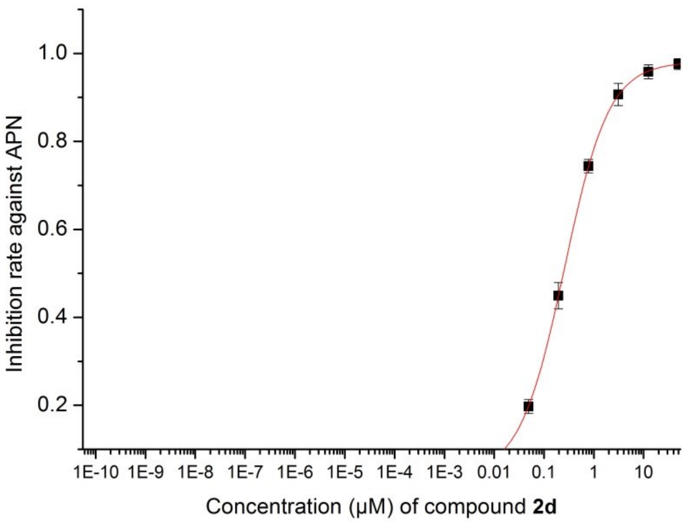
Inhibition rate against APN
1.0
0.8
0.6
0.4
0.2
1E-10 1E-9 1E-8 1E-7 1E-6 1E-5 1E-4 1E-3 0.01 0.1 1 10
Concentration (μM) of compound **2d**

F

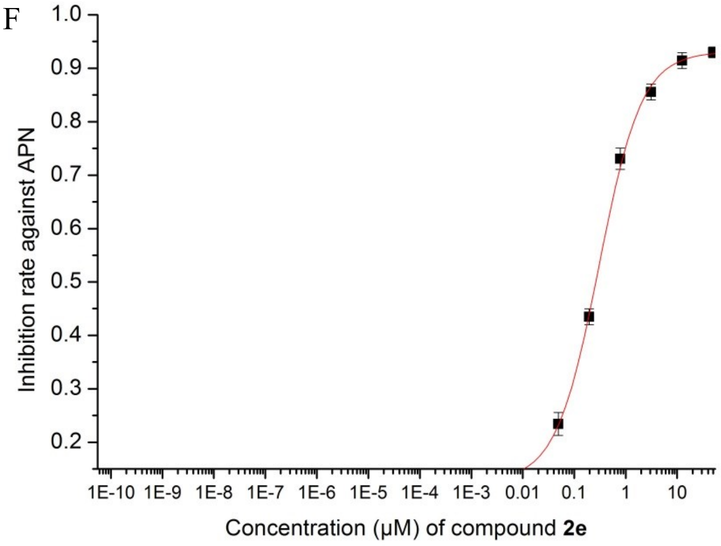
Inhibition rate against APN
1.0
0.9
0.8
0.7
0.6
0.5
0.4
0.3
0.2
1E-10 1E-9 1E-8 1E-7 1E-6 1E-5 1E-4 1E-3 0.01 0.1 1 10
Concentration (μM) of compound **2e**

G

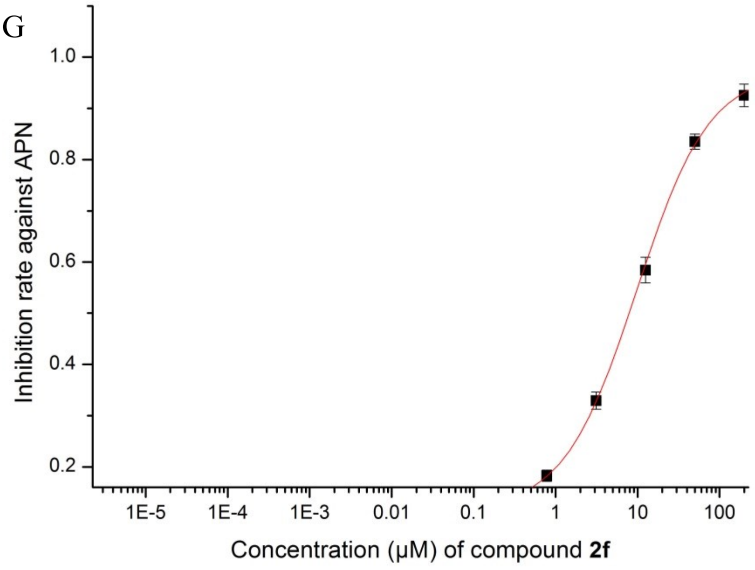
Inhibition rate against APN
1.0
0.8
0.6
0.4
0.2
1E-5 1E-4 1E-3 0.01 0.1 1 10 100
Concentration (μM) of compound **2f**

H

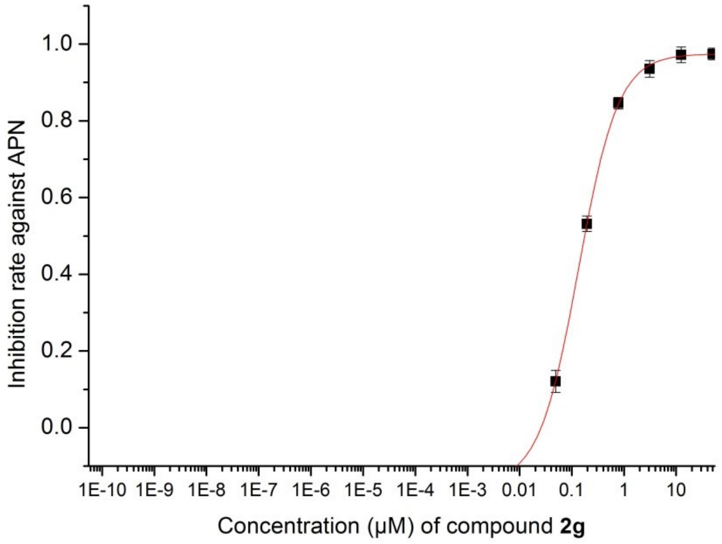
Inhibition rate against APN
1.0
0.8
0.6
0.4
0.2
0.0
1E-10 1E-9 1E-8 1E-7 1E-6 1E-5 1E-4 1E-3 0.01 0.1 1 10
Concentration (μM) of compound **2g**

I

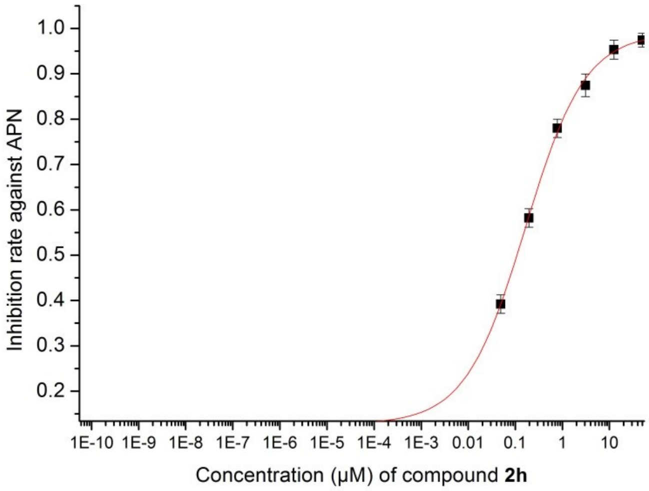
Inhibition rate against APN
1.0
0.9
0.8
0.7
0.6
0.5
0.4
0.3
0.2
1E-10 1E-9 1E-8 1E-7 1E-6 1E-5 1E-4 1E-3 0.01 0.1 1 10
Concentration (μM) of compound **2h**

J

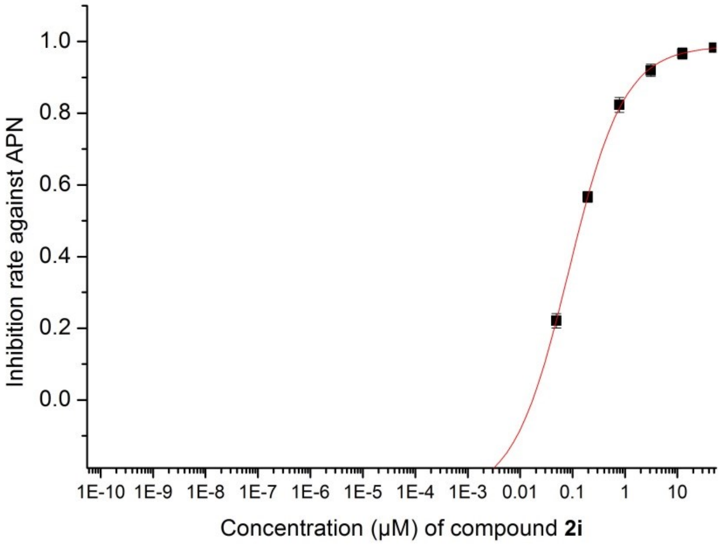
Inhibition rate against APN
1.0
0.8
0.6
0.4
0.2
0.0
1E-10 1E-9 1E-8 1E-7 1E-6 1E-5 1E-4 1E-3 0.01 0.1 1 10
Concentration (μM) of compound **2i**

K

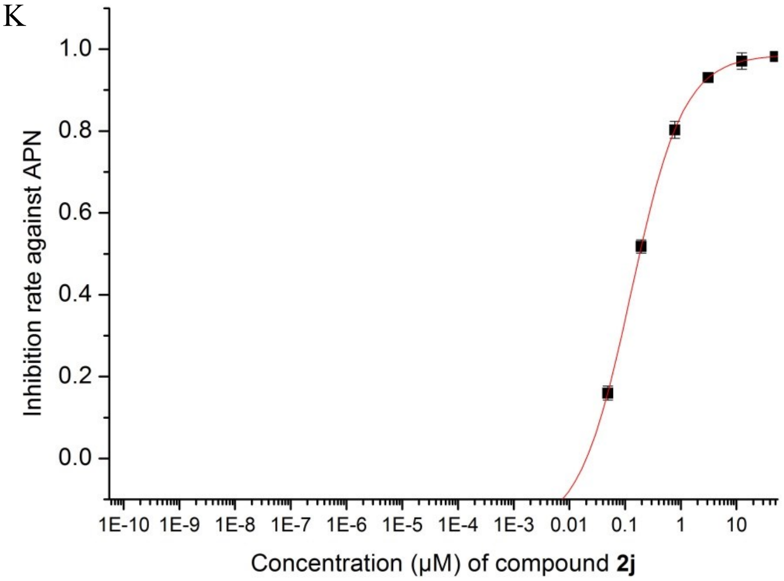

Inhibition rate against APN
1.0
0.8
0.6
0.4
0.2
0.0
1E-10 1E-9 1E-8 1E-7 1E-6 1E-5 1E-4 1E-3 0.01 0.1 1 10
Concentration (μM) of compound **2j**

L

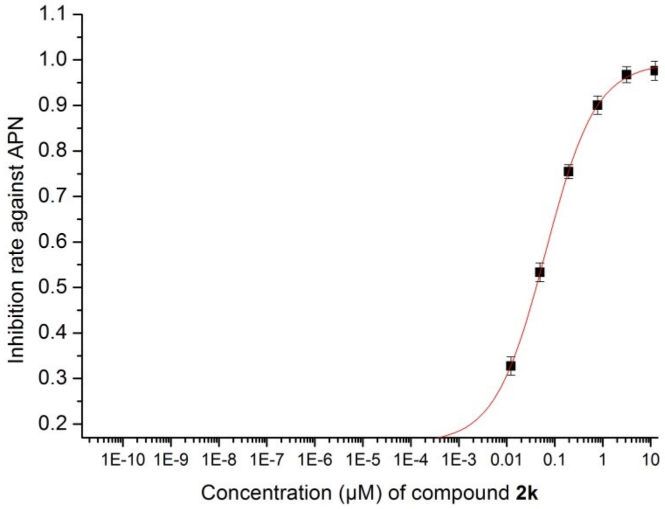

Inhibition rate against APN
1.1
1.0
0.9
0.8
0.7
0.6
0.5
0.4
0.3
0.2
1E-10 1E-9 1E-8 1E-7 1E-6 1E-5 1E-4 1E-3 0.01 0.1 1 10
Concentration (μM) of compound **2k**

M

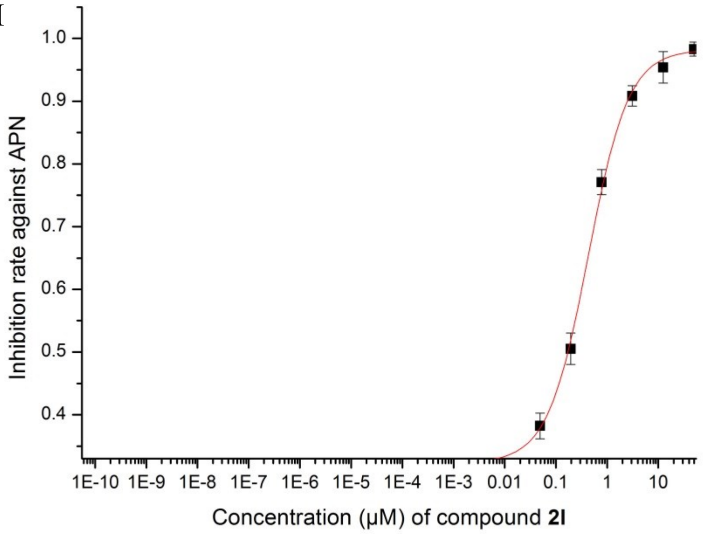

Inhibition rate against APN
1.0
0.9
0.8
0.7
0.6
0.5
0.4
1E-10 1E-9 1E-8 1E-7 1E-6 1E-5 1E-4 1E-3 0.01 0.1 1 10
Concentration (μM) of compound **2l**

N

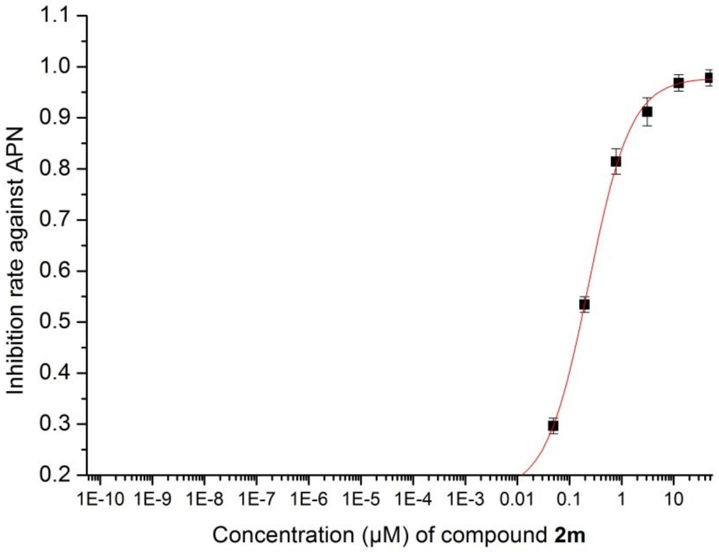
Inhibition rate against APN
1.1
1.0
0.9
0.8
0.7
0.6
0.5
0.4
0.3
0.2
1E-10 1E-9 1E-8 1E-7 1E-6 1E-5 1E-4 1E-3 0.01 0.1 1 10
Concentration (μM) of compound **2m**

O

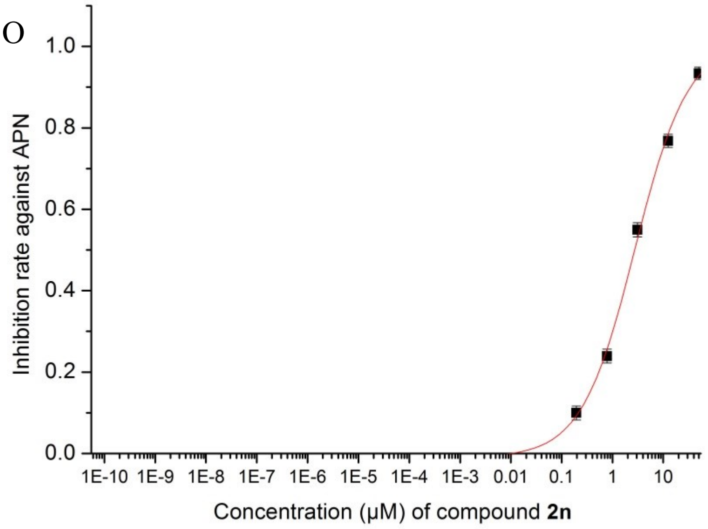
Inhibition rate against APN
1.0
0.8
0.6
0.4
0.2
0.0
1E-10 1E-9 1E-8 1E-7 1E-6 1E-5 1E-4 1E-3 0.01 0.1 1 10
Concentration (μM) of compound **2n**

P

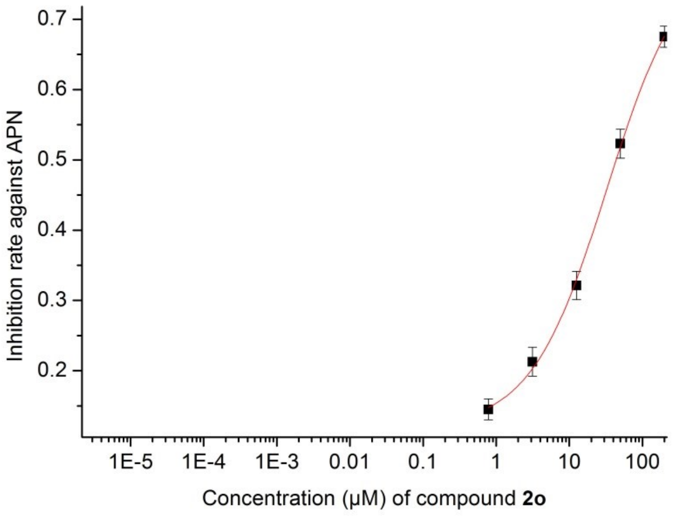
Inhibition rate against APN
0.7
0.6
0.5
0.4
0.3
0.2
1E-5 1E-4 1E-3 0.01 0.1 1 10 100
Concentration (μM) of compound **2o**

Q

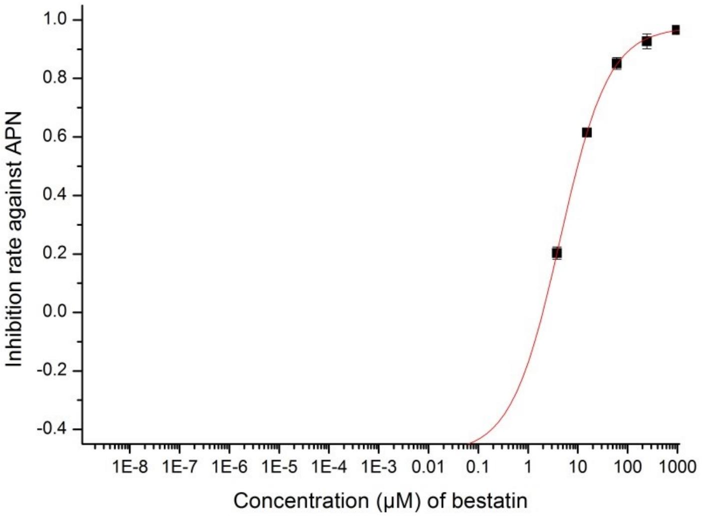

Figure S1. The IC_{50} graphs of compounds against APN. The pictures A-Q was for compounds **1**, **2a-2o** and bestatin, respectively. The bars indicate means ± SD (n = 3)
